# DYRK-family kinases regulate *Candida albicans* morphogenesis and virulence through the Ras1/PKA pathway

**DOI:** 10.1128/mbio.02183-23

**Published:** 2023-11-28

**Authors:** Jessie MacAlpine, Zhongle Liu, Saif Hossain, Luke Whitesell, Nicole Robbins, Leah E. Cowen

**Affiliations:** 1 Department of Molecular Genetics, University of Toronto, Toronto, Ontario, Canada; Duke University Hospital, Durham, North Carolina, USA

**Keywords:** *Candida albicans*, Yak1 kinase, DYRK, Ras1/PKA, morphogenesis, dermatitis, fungal pathogen

## Abstract

*Candida albicans* is an opportunistic human fungal pathogen that causes common superficial mycoses in healthy populations and invasive disease in immunocompromised individuals. The fungus employs several virulence traits to cause disease in humans, including its ability to transition from yeast to filamentous morphologies. Previous work found that genetic or pharmacological inhibition of the dual-specificity tyrosine-phosphorylation regulated kinase (DYRK) Yak1 blocks *C. albicans* morphogenesis and biofilm formation. Here, we expand on this work to provide mechanistic insights into how Yak1 governs this important virulence trait. First, we establish that Yak1 acts either downstream or parallel to protein kinase A (PKA) to regulate filamentation in response to diverse inducing cues. Furthermore, its importance in governing filamentous growth is dependent on core transcriptional regulators, such as Efg1 and Flo8. We also report that hyperactivation of the Ras1/cAMP/PKA pathway under elevated, host-relevant concentrations of CO_2_ bypasses the requirement for Yak1 in the yeast-to-filament transition. Seeking to identify the factor(s) that mediates filamentation under elevated CO_2_, we found that in a *yak1-*mutant background, homozygous deletion of a predicted, but previously uncharacterized DYRK, *POM1,* blocks filamentation. Finally, we demonstrate that Yak1 is required for morphogenesis in a dermatitis model of *C. albicans* infection and that pharmacological inhibition of Yak1 with a beta-carboline also attenuates filamentation in the dermal tissue. Overall, this work characterizes the role of Yak1 in regulating *C. albicans* morphogenesis, identifies an undescribed role for Pom1 in the yeast-to-filament transition, and suggests that inhibition of Yak1 may serve as a therapeutic strategy to combat *C. albicans* dermatitis.

**IMPORTANCE:**

*Candida albicans* is an opportunistic human fungal pathogen that frequently causes life-threatening infections in immunocompromised individuals. To cause disease, the fungus employs several virulence traits, including its ability to transition between yeast and filamentous states. Previous work identified a role for the kinase Yak1 in regulating *C. albicans* filamentation. Here, we demonstrate that Yak1 regulates morphogenesis through the canonical cAMP/PKA pathway and that this regulation is environmentally contingent, as host-relevant concentrations of CO_2_ bypass the requirement of Yak1 for *C. albicans* morphogenesis. We show a related kinase, Pom1, is important for filamentation in the absence of Yak1 under these host-relevant conditions, as deletion of both genes blocked filamentous growth under all conditions tested. Finally, we demonstrate that Yak1 is required for filamentation in a mouse model of *C. albicans* dermatitis using genetic and pharmacological approaches. Overall, our results expand our understanding of how Yak1 regulates an important virulence trait in *C. albicans*.

## INTRODUCTION

The fungal kingdom is extraordinarily diverse, encompassing millions of species that range from some of the largest organisms on Earth to single-cell microbes. For many fungi, the ability to sense and respond to environmental cues and transition between distinct morphologies has a profound impact on their biology, including the ability to reproduce and acquire nutrients (1–3). For human fungal pathogens, morphological plasticity also has important implications for the ability to invade tissues and evade host immune responses (1). One of the most common human fungal pathogens is *Candida albican*s, a commensal organism contributing to the normal mucosal microbiota in approximately 50% of healthy humans (4). Unfortunately, when the stable host-fungus relationship is disrupted, this organism frequently causes superficial infections, as well as life-threatening systemic disease in immunocompromised individuals, with mortality rates exceeding 40% even with therapeutic intervention (5). Poor clinical outcome for invasive candidiasis is in large part due to the limited number of available antifungal agents and the increasing prevalence of antifungal resistance (6). Thus, to combat this opportunistic pathogen, it is important to better understand the mechanisms enabling its transition between commensal- and disease-causing states and how these might be targeted with new antifungal agents.

The pathogenic prowess of *C. albicans* is dependent on a repertoire of virulence traits, including the secretion of hydrolytic enzymes, the production of the fungal toxin candidalysin, the capacity to form intrinsically drug-resistant biofilms, and the ability to undergo morphogenesis (1, 7, 8). The transition of *C. albicans* between yeast and filamentous morphologies (hyphal morphogenesis) is important for virulence, as the majority of genetic mutants locked in either state exhibit reduced pathogenicity in mouse models of infection (9–12). The transition can be induced through several environmental factors, which activate distinct signal transduction pathways (1). Perhaps the best-characterized signaling pathway responsible for regulating the yeast-to-filament transition is the Ras1/cyclic-AMP (cAMP)/protein kinase A (PKA) pathway (13, 14). This cascade involves the GTPase Ras1, which stimulates the adenylate cyclase Cyr1 to produce cAMP. This small molecule then relieves negative regulation of PKA by its regulatory subunit Bcy1, allowing the two catalytic subunits, Tpk1 and Tpk2, to phosphorylate and activate the transcription factor Efg1. PKA can also signal through other transcription factors that positively and negatively regulate the yeast-to-filament transition, including Ume6, Brg1, Tec1, Flo8, and Nrg1 (15, 16).

In addition to the Ras1/cAMP/PKA pathway, several other signal transduction cascades have been implicated in *C. albicans* morphogenesis. The mitogen-activated protein kinase (MAPK) pathway involves activation of the GTPase Cdc42, which signals through a downstream MAPK cascade involving Ste11, Hst7, and Cek1/Cek2, ultimately activating the transcription factor Cph1 (17). In contrast to the cAMP-PKA pathway, many components of the MAPK pathway are dispensable for filamentation under certain inducing cues (18). Additional regulators implicated in governing filamentation in response to diverse environmental stimuli include the target of rapamycin (TOR) kinase, as well as protein kinase C (Pkc1) (19–21). Finally, previous work identified a small molecule that blocks *C. albicans* morphogenesis through the inhibition of the kinase, Yak1(22), which is known to regulate *C. albicans* filamentation in response to select inducing cues (23). However, little is known about the mechanism(s) by which Yak1 regulates this important virulence trait.

Yak1 is a member of an evolutionarily conserved family of Ser/Thr protein kinases known as dual-specificity tyrosine-phosphorylation regulated kinases (DYRKs), which are characterized by a YXY motif in the activation loop of the catalytic domain (24, 25). Autophosphorylation on the second tyrosine residue in the YXY motif is essential for Yak1 stability and activity in the model yeast *Saccharomyces cerevisiae* (26). While our understanding of the signaling circuitry through which Yak1 functions in *C. albicans* is limited, previous work in *S. cerevisiae* originally characterized Yak1 as a suppressor of the lethality associated with loss of function of either Ras1 or PKA, implicating Yak1 as a negative regulator of PKA-regulated phenotypes such as growth in glucose, heat sensitivity, and pseudohyphal growth (27, 28). Follow-up studies confirmed that PKA phosphorylates Yak1 in *S. cerevisiae,* both *in vitro* and *in cellulo,* to prevent its nuclear translocation and activation (29, 30). Under stress conditions or glucose starvation, PKA is inhibited, thus alleviating this negative regulation, and allowing Yak1 to translocate to the nucleus and activate downstream transcription factors important for stress responses. Interestingly, phenotypic analyses of *yak1* loss-of-function mutants in *C. albicans* suggest divergence in Yak1 regulation between the fungal pathogen and model yeast (23). Pharmacological or genetic inhibition of Yak1 function inhibits filamentation, akin to loss of function of PKA, implicating both kinases as positive regulators of morphogenesis (22). Thus, defining the mechanism(s) by which Yak1 regulates core signal transduction pathways is needed to better understand how this unusual dual-specificity kinase supports virulence in *C. albicans*.

Here, we reveal that Yak1 plays an important role in orchestrating the yeast-to-filament transition in *C. albicans* in an environmentally-contingent manner. Genetic epistasis analysis indicates that Yak1 acts downstream or parallel to PKA in *C. albicans* and is dependent on core transcription factors that regulate morphogenesis, including Efg1 and Flo8. While Yak1 plays a pivotal role in hyphal morphogenesis in response to multiple inducing cues, it is dispensable for filamentation in the presence of physiological concentrations of CO_2_, due to hyperactivation of the Ras1/cAMP/PKA pathway. Interestingly, deletion of another predicted DYRK-encoding gene in *C. albicans*, *POM1*, in a background lacking *YAK1* blocks filamentation under physiological CO_2._ This finding suggests that these kinases act in parallel to regulate morphogenesis in an environmentally-contingent manner and highlights a previously undescribed role for Pom1 in regulating *C. albicans* filamentation. Finally, we find that Yak1 is required for filamentation and dermal invasion in a mouse model of *Candida* dermatitis. Overall, this work deepens our understanding of the circuitry through which Yak1 regulates *C. albicans* morphogenesis under host-relevant conditions, implicates Pom1 as a regulator of filamentation, and unveils Yak1 as a key regulator of virulence in a mouse model of *C. albicans* dermatitis.

## RESULTS

### Function of Yak1 as a core regulator of hyphal morphogenesis depends on a conserved tyrosine residue in its kinase domain

**Fig 1:**
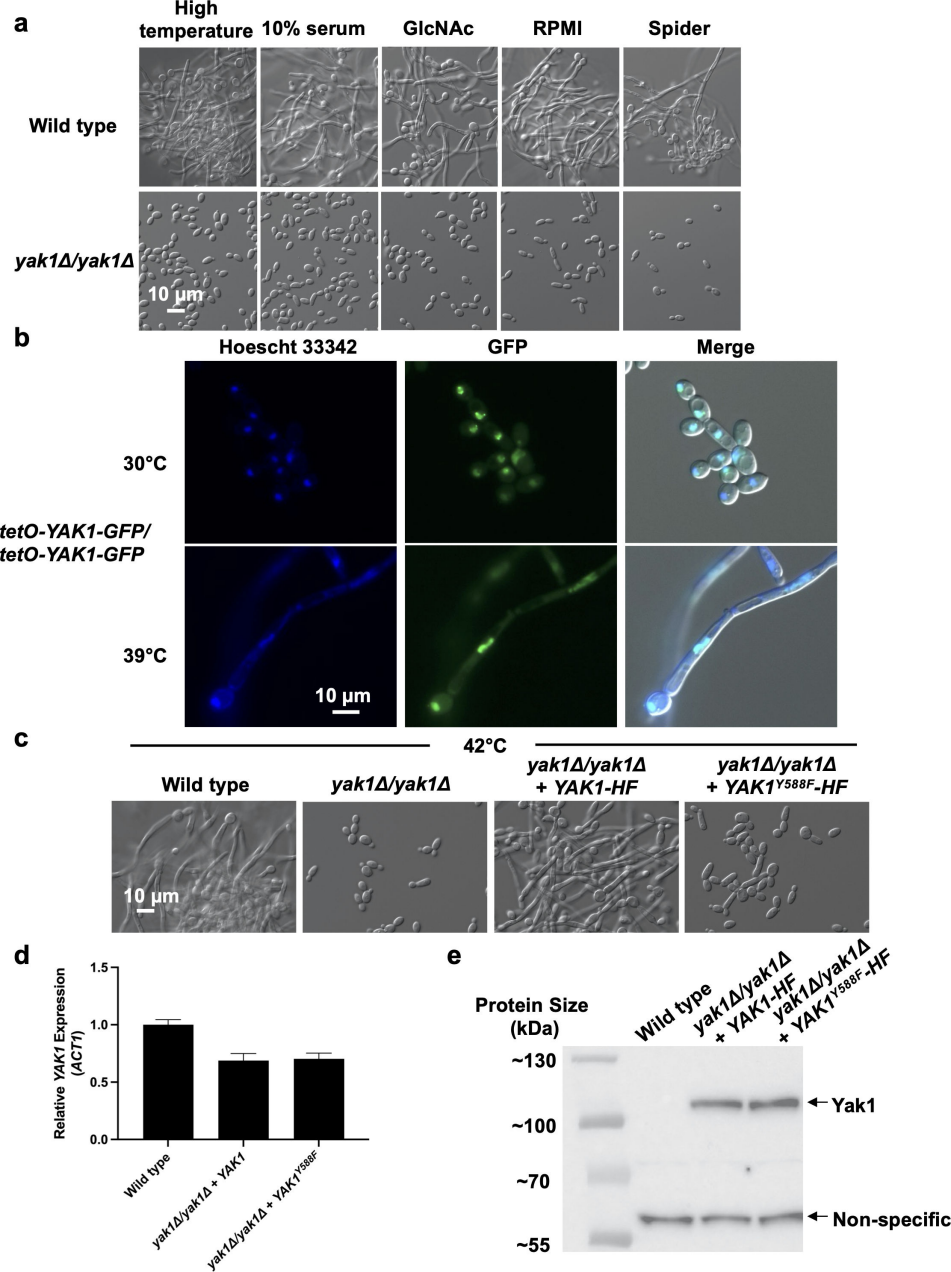
Yak1 is a core regulator of hyphal morphogenesis in *C. albicans*. (a) Yak1 is required for *C. albicans* morphogenesis in response to diverse inducing cues, including growth at igh temperature (42°C, 4 hours), in 10% serum (37°C, 4 hours), in N-acetyl-glucosamine (GlcNAc; 5 mM, 37°C, 4 hours), in RPMI (37°C, 4 hours), and in Spider medium (37°C, 4 hours). Light microscopy images are representative of two biological replicates. (b) Yak1 is localized to the nucleus under both basal and filament-inducing conditions. Cells were grown for 3 hours at either 30°C or 39°C, as indicated. Cells were collected, washed twice with PBS, and fixed with 4% paraformaldehyde. Cells were then stained with 5 µg/mL of the nuclear dye, Hoescht 33342, and imaged using light and fluorescence microscopy. Data are representative of two biological replicates. (c) Y588 is required for Yak1 function in *C. albicans*. In response to the inducing cue of high temperature (42°C), complementation of a *yak1* homozygous-deletion mutant with one allele of *YAK1* rescues morphogenesis, but complementation with one allele of the phospho-ablated *YAK1*^Y588F^ does not rescue the defect. Cells were grown in YPD at 42°C for 4 hours to induce morphogenesis. Light microscopy images are representative of two biological replicates. (d) Mutation of *YAK1* does not alter its expression. Cells were grown at 30°C for 3 hours. Transcript levels relative to the wild-type control were measured by RT-qPCR and normalized to *ACT1*. Data are presented as mean of technical quadruplicates. Measure of center represents the mean of the data, and error bars represent the standard error of the mean. Results are representative of two biological replicates. (e) Cellular level of Yak1^Y588F^ is comparable to that of wild-type Yak1 protein. Cells were grown at 30°C for 4 hours and then protein was extracted under denaturing conditions. Samples were run on an 8% SDS-PAGE gel and probed with an anti-FLAG antibody to assess relative protein levels. Data are representative of two biological replicates.

In *C. albicans,* the yeast-to-filament transition is regulated by multiple signaling pathways, some of which play pivotal roles in response to many inducing cues, while others are more selective (1). Previous studies have shown that a *yak1* homozygous-deletion mutant is blocked in hyphal morphogenesis upon exposure to high temperature (42°C), in the presence of 10% serum at 37°C, in Lee’s medium at 37°C, and on solid agar that induces filamentation, including RPMI agar and YPD agar supplemented with 20% serum (22, 23). Thus, we began by testing the importance of Yak1 in regulating hyphal morphogenesis in response to other inducing cues. While a wild-type control underwent robust filamentation in all conditions tested, a *yak1* homozygous-deletion mutant was blocked in morphogenesis in response to high temperature, 10% serum, N-acetyl-glucosamine, RPMI medium, and Spider medium (Fig. 1a). Thus, Yak1 is an important regulator of filamentation in response to diverse environmental stimuli.

In *S. cerevisiae,* Yak1 remains in the cytoplasm until it becomes activated, at which point it translocates to the nucleus to stimulate transcriptional changes in response to environmental cues (29). To determine whether differential localization of Yak1 occurs in *C. albicans* under filament-inducing versus non-inducing conditions, we generated a strain in which both alleles of *YAK1* were modified to encode a C-terminal green fluorescent protein (GFP) tag and placed under the control of the strong *tetO* promoter to enable visualization of the modified protein. The ability of the strain to filament at elevated temperatures confirmed the functionality of this tagged protein (Fig. 1b). We found that Yak1-GFP was constitutively localized to the nucleus in *C. albicans* under both basal and filament-inducing conditions as detected by the co-localization of GFP with a Hoechst 33342 nuclear stain (Fig. 1b). Yak1 is a serine/threonine protein kinase whose stability and activity in *S. cerevisiae* are modulated by autophosphorylation at a conserved tyrosine residue (29). Therefore, we tested whether this residue was also important for the function and stability of *C. albicans* Yak1. To do so, we complemented a *yak1* homozygous-deletion mutant with either a wild-type allele that encodes a C-terminal His_6_-FLAG_3_ tag (*YAK1-HF*) or with an identically tagged allele in which the conserved tyrosine residue (*C. albicans* Y588, *S. cerevisiae* Y530 Fig. S1, black box) was changed to phenylalanine to generate a phospho-ablation mutant (*YAK1^Y588F^-HF*). Complementation of the *yak1Δ/yak1Δ* mutant with the wild-type *YAK1-HF* allele restored filamentation at 42°C, confirming that the tagged protein remained functional (Fig. 1c). In contrast, the Y588F substitution resulted in a defect in *C. albicans* morphogenesis, akin to what was observed in a *yak1Δ/yak1Δ* control, indicating that this residue is required for Yak1 to function in *C. albicans* (Fig. 1c). To address whether the Y588F substitution disrupts Yak1 function by decreasing gene expression or protein stability, we monitored transcript expression and protein level in the strains through RT-qPCR and western blot, respectively. Interestingly, in both complemented strains, transcript and protein levels were comparable to one another (Fig. 1d and e), indicating that substitution of this key tyrosine residue compromises Yak1 function without affecting expression or protein levels. This finding diverges sharply from the biology reported for *S. cerevisiae* and human cells, where modification at this site destabilizes the protein (26, 31, 32). Overall, these results reveal a mode of action for *C. albicans* Yak1 distinct from that previously reported in other organisms and establishes this kinase as a core regulator of hyphal morphogenesis that resides constitutively in the nucleus and relies on a conserved tyrosine residue in its activation loop for biological function, but not protein stability.

### Yak1 acts downstream or parallel to PKA in *C. albicans* to regulate morphogenesis

In *S. cerevisiae*, PKA phosphorylates Yak1 to inhibit its activity (29, 30). Given this mode of regulation in the model yeast, as well as the observation that both PKA and Yak1 are important regulators of morphogenesis in *C. albicans* (Fig. 1a) (13, 23), we predicted that Yak1 would act within the canonical Ras1/cAMP/PKA filamentation-regulatory pathway in *C. albicans*. Two catalytic subunits, Tpk1 and Tpk2, are found in the PKA holoenzyme. Homozygous deletion of genes encoding either subunit results in a defect in morphogenesis (33), with a *tpk2* homozygous-deletion mutant exhibiting a more severe block in filamentation in a variety of liquid conditions, but not on solid media.

**Fig 2:**
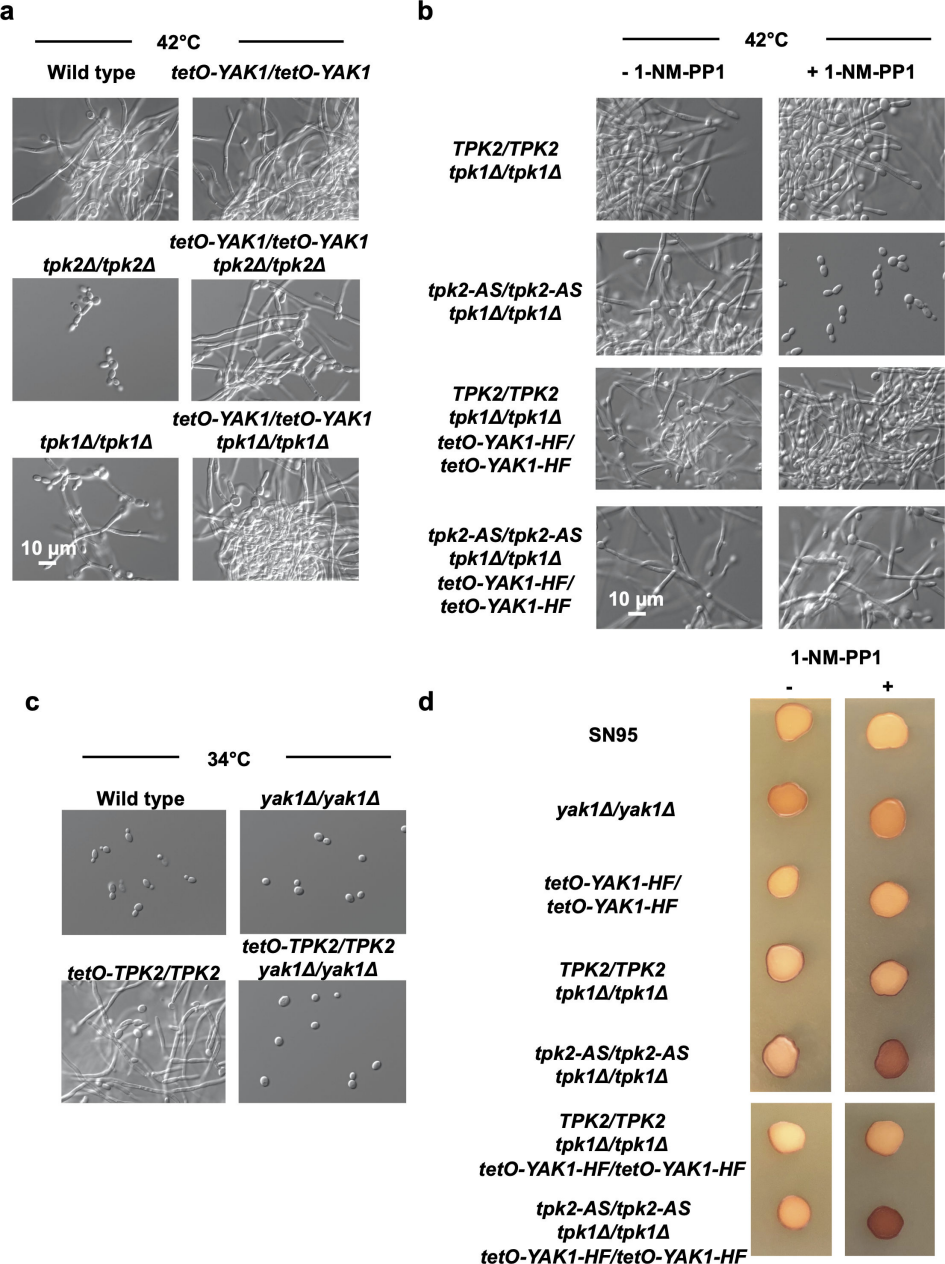
Yak1 acts downstream or parallel to PKA to induce *C. albicans* filamentation. (a) Homozygous deletion of genes encoding either of the PKA subunits, *TPK1* or *TPK2*, results in a defect in *C. albicans* morphogenesis. Overexpression of *YAK1* driven by a *tetO* promoter rescues the filamentation defect in both deletion-strain backgrounds. Cells were grown in YPD at 42°C for 4 hours. Light microscopy images are representative of two biological replicates. (b) Overexpression of *YAK1* rescues the defect in filamentation observed with the inhibition of PKA activity. One PKA subunit gene, *TPK1,* was deleted and the remaining subunit gene, *TPK2,* was replaced with an allele encoding an analog-sensitive (AS) version of the kinase; treatment of this strain with the ATP analog 1-NM-PP1 selectively inhibits the kinase activity of *TPK2*. Cells were grown in YPD at 42°C in the absence or presence of 2.5 µM 1-NM-PP1 for 4 hours. Light microscopy images are representative of two biological replicates. (c) Overexpression of *TPK2* driven by a *tetO* promoter results in a hyper-filamentous phenotype under basal conditions. Cells were grown at 34°C for 6 hours. Homozygous deletion of *YAK1* in the *tetO-TPK2/TPK2* background restores wild-type morphology. Light microscopy images are representative of two biological replicates. (d) Yak1 does not affect PKA regulation of glycogen storage. All strains were grown on YPD agar either with or without 2.5 µM 1-NM-PP1 for 2 days. The plate was then inverted over iodine crystals for 5 minutes to stain for glycogen content. Overexpression of *YAK1* does not rescue the increase in glycogen accumulation observed in a strain of *C. albicans*, where *TPK1* is deleted and *TPK2* encodes an analog-sensitive version of the kinase that can be inhibited by the ATP analog, 1-NM-PP1. Images are representative of two biological replicates.

To determine whether Yak1 acts downstream of Tpk1 and Tpk2, we first performed a genetic epistasis analysis. We constructed strains of *C. albicans* in which *YAK1* is overexpressed in a wild-type, *tpk1Δ/tpk1Δ,* or *tpk2Δ/tpk2Δ* genetic background by placing both alleles under the control of the strong *tetO* promoter (Fig. S2a). While homozygous deletion of *TPK1* impaired filamentation and deletion of *TPK2* completely blocked filamentation induced by elevated temperature, overexpression of *YAK1* rescued the defect in both the *tpk1* and *tpk2* homozygous-deletion backgrounds (Fig. 2a). As a complementary approach, we next utilized a strain of *C. albicans* in which one catalytic subunit gene, *TPK1,* is deleted, and the remaining subunit gene, *TPK2,* is replaced with an analog-sensitive version of the allele (34). This analog-sensitive allele encodes a mutation of the kinase gatekeeper residue, which renders the kinase sensitive to the bulky ATP analog, C3-1′-naphthyl-methyl PP1 (1-NM-PP1) (35, 36). Upon addition of 1-NM-PP1, PKA activity could be rapidly inhibited in this strain, blocking filamentation (Fig. 2b). Overexpression of *YAK1* in this mutant background rescued filamentation despite the presence of 1-NM-PP1 (Fig. 2b; Fig. S2b), providing additional evidence that Yak1 may act downstream of PKA in *C. albicans*. Finally, to provide further genetic evidence supporting the hypothesis that Yak1 acts downstream of PKA, we generated a strain where *TPK2* is overexpressed in both wild-type and *yak1Δ/yak1Δ* genetic backgrounds (Fig. S2c). While overexpression of *TPK2* resulted in a hyper-filamentous phenotype at 34°C, deletion of *YAK1* blocked filamentation under these conditions (Fig. 2c). Overall, these experiments support a model in which Yak1 acts downstream of PKA or it acts in a parallel pathway that is both required for filamentation even upon hyperactivation of PKA and is sufficient to drive filamentation in the absence of PKA.

Next, we wanted to learn whether Yak1 acts downstream of PKA to modulate diverse biological responses or if it is specific to the yeast-to-filament transition. To do so, we once again used the strain where PKA activity can be rapidly inhibited through the addition of the ATP analog 1-NM-PP1 (*tpk2-AS/tpk2-AS tpk1Δ/tpk1Δ*). A well-described consequence of PKA inhibition is an increase in intracellular glycogen storage, which can be evaluated by monitoring colony color following exposure to iodine vapor (37). For the *tpk2-AS/tpk2-AS tpk1Δ/tpk1Δ* strain, supplementation of the agar medium with 1-NM-PP1 resulted in a change from light beige colony color to dark brown colony color after iodine treatment, indicative of increased glycogen accumulation upon inhibition of PKA (Fig. 2d). However, overexpression of *YAK1* did not rescue the glycogen hyperaccumulation phenotype, as colonies still appeared dark brown after growth in the presence of 1-NM-PP1, similar to the result seen when *YAK1* was expressed at endogenous levels (Fig. 2d). These data suggest that Yak1 acts downstream or parallel to PKA to regulate *C. albicans* morphogenesis but does not play a role in other PKA-regulated phenotypes, such as glycogen accumulation.

### Mutation of predicted PKA phosphorylation sites alters Yak1 function

**Fig 3:**
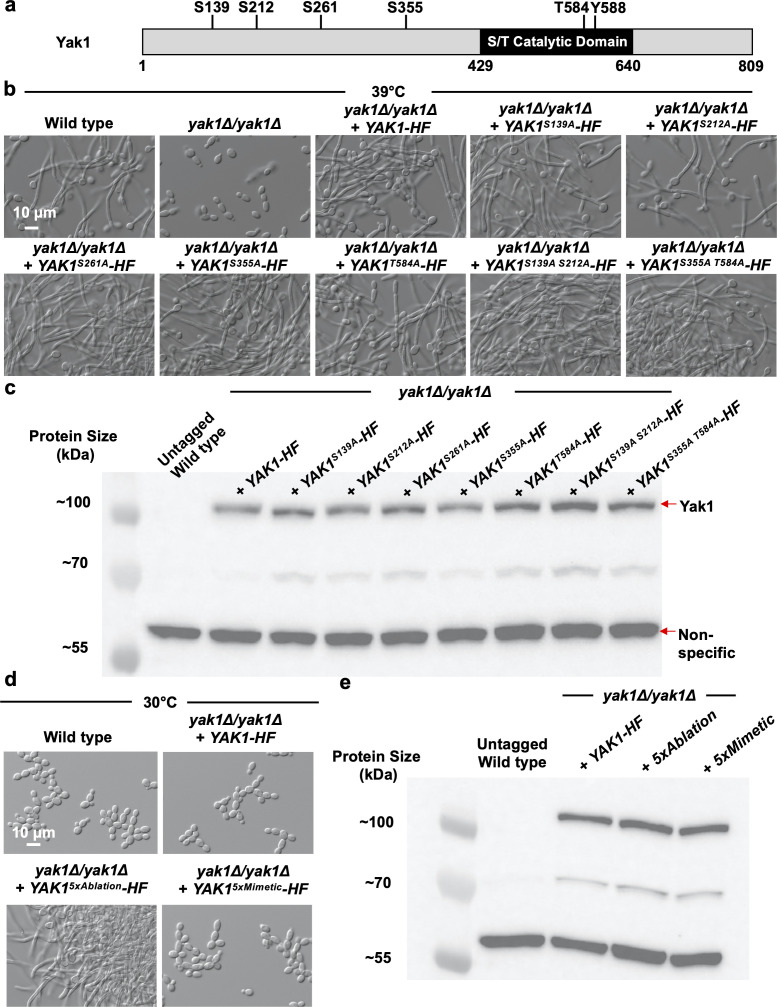
Mutation of predicted PKA phosphorylation sites in Yak1 does not inhibit its function. (a) Schematic of the five predicted PKA phosphorylation residues in *C. albicans* Yak1. (b) Phospho-ablation of predicted PKA phosphorylation sites S139, S212, S261, S355, or T584 does not affect the ability of *C. albicans* to undergo morphogenesis. Cells were grown at 39°C for 4 hours. Light microscopy is representative of two biological replicates. (c) Phospho-ablation of any of the five predicted PKA-phosphorylated residues in Yak1 does not alter protein level. Cells were grown at 30°C for 3 hours and then protein was extracted under denaturing conditions. Samples were fractionated on an 8% SDS-PAGE gel and probed with an anti-FLAG antibody. Western blot is representative of two biological replicates. (d) Phospho-ablation (5×Ablation-HF) of all five predicted PKA phosphorylation sites in Yak1 results in a hyper-filamentous phenotype. However, substitution with a phospho-mimetic residue at each of the five predicted sites in Yak1 (5×Mimetic-HF) does not alter *C. albicans* morphogenesis. Cells were grown at 30°C for 4 hours. Light microscopy is representative of two biological replicates. (e) Phospho-ablative or phospho-mimetic substitutions at all five predicted PKA phosphorylation sites in Yak1 do not alter protein level. Cells were grown at 30°C for 3 hours and then protein was extracted under denaturing conditions. Samples were fractionated on an 8% SDS-PAGE gel and probed with an anti-FLAG antibody. Western blot is representative of two biological replicates.

In *S. cerevisiae,* Yak1 is phosphorylated by PKA at conserved PKA-consensus sites to modulate its function. To determine if *C. albicans* PKA similarly regulates Yak1 function through direct phosphorylation, we analyzed five amino acid residues previously predicted to be phosphorylated by PKA in *C. albicans*: S139, S212, S261, S355, and T584 (Fig. 3a) (23, 38). To assess the functional importance of phosphorylating these residues, site-directed mutagenesis was used to construct five strains, each expressing a non-phosphorylatable alanine at one of the predicted sites as the sole source of Yak1. Substitution of serine or threonine for alanine did not affect the ability of *C. albicans* to undergo hyphal morphogenesis induced by elevated temperature, indicating that no single residue among the five sites examined is required for Yak1’s function (Fig. 3b). Importantly, the substitutions did not affect the expression of the corresponding mutant genes (Fig. S3a) or the cellular level of the mutant proteins (Fig. 3c). To examine if phospho-ablation of multiple residues was required to abrogate Yak1 function, we leveraged structural insights obtained from crystal structures of DYRK kinases in other organisms in addition to our previously reported computational model of *C. albicans* Yak1 structure (22). This analysis predicted that S139 and S212 were located in a predicted unstructured region of the protein, and S355 and T584 were predicted to be in structured, conserved regions of the protein. Thus, we generated Yak1 alleles that encoded phospho-ablation substitutions at both S139 and S212 or S355 and T584. However, phospho-ablation at these dual sites did not block *C. albicans* from filamenting at elevated temperature (Fig. 3b) or impact transcript or protein expression (Fig. S3a; Fig. 3c).

Finally, we asked whether mutating all five residues (S139, S212, S261, S355, and T584) simultaneously would impact the Yak1 function. To do so, we first investigated whether phospho-mimetic substitutions at all five residues (5×-Mimetic) would be sufficient to constitutively activate Yak1 and promote filamentous growth in the absence of an inducing cue. Surprisingly, despite similar transcript and protein expression as in the wild-type control, substitution of all five residues to either glutamate or aspartate did not induce filamentation in the absence of an inducing cue, and importantly did impair the strain from filamenting at elevated temperature (Fig. 3d and e; Fig. S3b and c). To assess whether phospho-ablation at all five sites collectively would impair Yak1 activity, we generated a 5×-phospho-ablation (5×-Ablation) mutant and monitored the phenotype of the strain under basal and filament-inducing conditions. Interestingly, phospho-ablation of all five sites in combination resulted in a hyper-filamentous phenotype under basal growth conditions (Fig. 3d), which was not due to increased transcript expression or protein accumulation (Fig. 3e; Fig. S3b). Notably, phospho-ablation at any of the individual sites or the combinations examined (S139A and S212A or S355A and T584A) did not result in a hyper-filamentous phenotype in the absence of an inducing cue (Fig. S3c). We confirmed that the constitutive filamentation phenotype was due to the expression of the 5×-Ablation encoding *YAK1* allele, as when we put it under the control of the *tetO* promoter, repression of this allele restored yeast growth under basal conditions (Fig. S3e).

Results with our site-directed mutant strains strongly suggest a very different mode of Yak1 regulation in *C. albicans* than previously described for *S. cerevisiae*, in which PKA-mediated phosphorylation of Yak1 is required for activation of its function (29). Further investigation will be required in the future to decipher the mechanism(s) by which PKA, either directly or indirectly, acts upon Yak1 to regulate its function. Several potential explanations for the disparity observed remain to be explored. These include but are not limited to (i) deciphering whether PKA indeed phosphorylates these five sites or whether PKA directly phosphorylates Yak1 at other residues important for its activity, (ii) investigating whether other kinases or phosphatases phosphorylated by PKA regulate Yak1 activity at the five residues examined, and (iii) determining whether dephosphorylation at the sites examined results in enhanced phosphorylation of Yak1 at the autophosphorylated Tyr residue (Y588), which drives Yak1 activity. Regardless, the results presented do identify five critical sites in Yak1, which affect its function such that their collective mutation has profound effects on *C. albicans* filamentation.

### Yak1 is dependent on core transcription factors to regulate *C. albicans* morphogenesis

**Fig 4:**
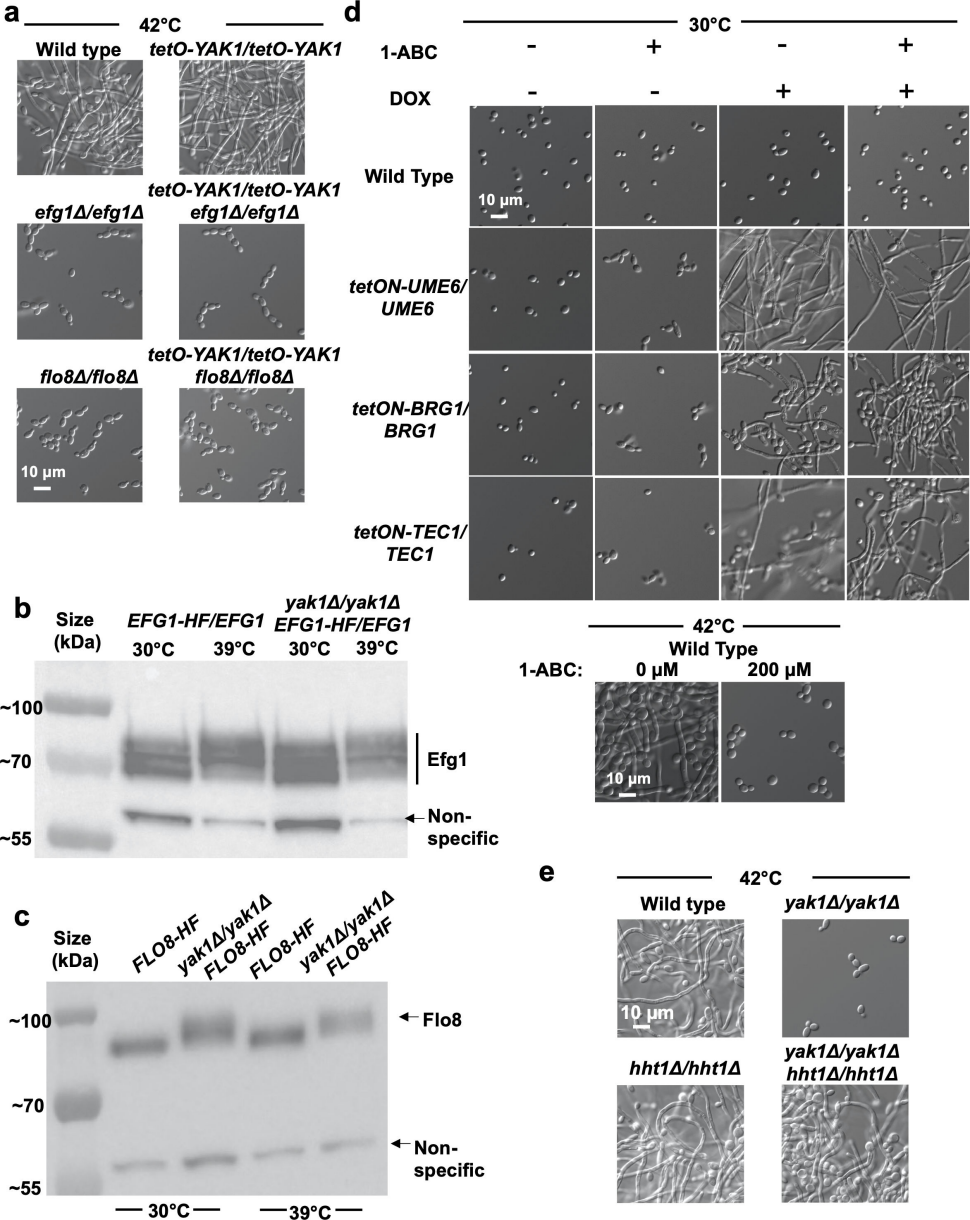
Yak1 is dependent on canonical transcription factors known to regulate *C. albicans* morphogenesis. (a) Deletion of *FLO8* or *EFG1* blocks filamentation, and overexpression of *YAK1* does not rescue this defect in either strain background. Strains were grown in RPMI with 10% serum at 37°C for 6 hours and visualized by microscopy. Images are representative of two biological replicates. (b) Migration of Efg1 during SDS-PAGE is unaffected by the deletion of *YAK1*. Strains were grown at either 30°C or 39°C for 3 hours, as indicated. Protein was extracted under denaturing conditions and an anti-FLAG antibody was used to assess the relative protein level and migration position via western blot analysis. Position of a non-specific immunoreactive band is indicated by an arrow. Data are representative of two biological replicates. (c) Deletion of *YAK1* results in reduced mobility of Flo8. Strains were grown at either 30°C or 39°C for 3 hours, as indicated. Protein was extracted under denaturing conditions, and an anti-FLAG antibody was used to assess migration position via western blotting. Position of a non-specific immunoreactive band is indicated by an arrow. Data are representative of two biological replicates. (d) At 30°C, overexpression of the transcription factors *UME6, BRG1,* or *TEC1* driven by a *tetON* promoter system induced by doxycycline (DOX, 10 µg/mL) drives filamentation in *C. albicans*. Treatment with the Yak1 inhibitor 1-acetyl-beta-carboline (1-ABC; 200 µM) does not block the mutant phenotype in any of the overexpression strains. Strains were grown in the absence or presence of DOX or 1-ABC for 8 hours at 30°C. To confirm that 1-ABC could inhibit filamentation under the conditions tested, a wild-type strain was grown in YPD at 42°C for 4 hours in the absence or presence of 200 µM 1-ABC. Light microscopy images are representative of two biological replicates. (e) Homozygous deletion of the histone H3 variant gene *HHT1* rescues the filamentation defect observed in a *yak1* homozygous-deletion mutant. Cells were grown at 42°C for 4 hours. Light microscopy images are representative of two biological replicates.

Given our genetic epistasis analyses mapping Yak1 downstream or parallel to PKA, we next wanted to determine if Yak1 is dependent on core transcription factors that regulate *C. albicans* morphogenesis. First, we determined whether Yak1 function is dependent on Efg1 and Flo8, transcription factors known to act downstream of PKA to positively regulate filamentation. Homozygous deletion of either *EFG1* or *FLO8* resulted in a complete block in *C. albicans* filamentation induced by elevated temperature (Fig. 4a). Overexpression of *YAK1* with the *tetO* promoter in either mutant background was unable to rescue the filamentation defect (Fig. 4a; Fig. S4a), indicating *YAK1* is dependent on both *EFG1* and *FLO8* and potentially acts upstream of these transcriptional regulators.

Subsequently, we examined whether Yak1 altered the phosphorylation status of either Efg1 or Flo8 under filament-inducing and non-inducing conditions (39). To do so, we leveraged epitope-tagged versions of either Efg1 or Flo8 and monitored the mobility of the proteins using western blot analysis. Cultures grown under inducing conditions (39°C) exhibit a shift of Efg1 relative to non-inducing conditions (30°C), but homozygous deletion of *YAK1* did not alter the cellular level or mobility of Efg1 at either temperature relative to a wild-type control (Fig. 4b). In contrast, the deletion of *YAK1* resulted in an upward mobility shift for Flo8 at both 30°C and 39°C relative to the wild type (Fig. 4c). Notably, the mobility of Flo8 was identical under inducing (39°C) and non-inducing conditions (30°C), suggesting that the shift seen upon *YAK1* deletion was not indicative of activation of the transcription factor. Furthermore, localization of Flo8 did not change upon homozygous deletion of *YAK1* under basal conditions (Fig. S4b). The reduced mobility of Flo8 associated with homozygous deletion of *YAK1* suggests a potential increase in phosphorylation of the protein, and previous work has indicated that phosphorylation of Flo8 inhibits its activity (40). Therefore, loss of Yak1 may increase the phosphorylation of Flo8 to reduce its activity, potentially through the activity of another kinase or phosphatase. Overall, these results suggest that *YAK1* deletion indirectly alters post-translational modification(s) of Flo8 with additional studies needed to identify the modifications and their functional consequences.

With genetic epistasis experiments suggesting that Yak1 is dependent on both Efg1 and Flo8, we wanted to determine if the kinase was dependent on other transcription factors to mediate hyphal morphogenesis. Using a previously published collection of overexpression strains in which a gene of interest is placed under the control of a *tetON* promoter (41), *UME6, BRG1,* and *TEC1* were overexpressed by the addition of the tetracycline analog, doxycycline (DOX) to the culture medium. Overexpression of all three transcription factors resulted in filamentation in the absence of an inducing cue (YPD, 30°C) (Fig. 4d). To assess whether inhibition of Yak1 blocked filamentation caused by overexpression of these transcription factors, we used the Yak1 inhibitor, 1-acetyl-beta-carboline (1-ABC), which blocks *C. albicans* morphogenesis (Fig. 4d, bottom panel) (22). Exposure to 1-ABC was unable to block filamentation of the *tetON-UME6/UME6, tetON-BRG1/BRG1,* or *tetON-TEC1/TEC1* strains (Fig. 4d), indicating overexpression of each of these transcription factors is sufficient to drive filamentation even in the absence of Yak1.

Thus far, results indicated that overexpression of several different positive regulators of filamentation could overcome the requirement for Yak1 and induce *C. albicans* morphogenesis in the absence of the kinase. Given the broad range of transcription factors involved, we hypothesized that Yak1 might act at the level of chromatin structure to increase the accessibility of relevant promoter elements and facilitate their transactivation. In this scenario, overexpression of transcription factors to a level that induces constitutive filamentation would bypass the need for Yak1 activity. Consistent with this hypothesis, we identified a consensus DYRK phosphorylation site in the histone H3 variant encoded by *HHT1* in *C. albicans* (42). Hht1 is a structural constituent of nucleosomes in the chromatin of *C. albicans*. While the molecular mechanism through which loss of *HHT1* alters chromatin accessibility is not well understood, homozygous deletion of *HHT1* results in differential gene expression of approximately one-fifth of all *C. albicans* genes, highlighting global changes in transcriptional activity, which collectively result in a constitutively filamentous phenotype (43). To begin testing this chromatin-based hypothesis, we generated a mutant strain with both copies of *YAK1* and *HHT1* deleted. We postulated that if deletion of *YAK1* results in loss of Yak1-mediated phosphorylation of Hht1 and limits access to the DNA of transcription factors, which promote the yeast-to-filament transition, deletion of *HHT1* should restore chromatin access and overcome the filamentation defect in a *yak1* homozygous-deletion mutant. Indeed, homozygous deletion of *HHT1* restored hyphal morphogenesis in response to elevated temperature in a *yak1* homozygous-deletion strain (Fig. 4e). Therefore, Yak1 is dependent on several transcription factors to modulate the morphogenetic transcriptional program, perhaps by influencing chromatin dynamics through mechanisms that will be interesting to investigate in future work.

### Yak1 regulates *C. albicans* morphogenesis in an environmentally-contingent manner

**Fig 5:**
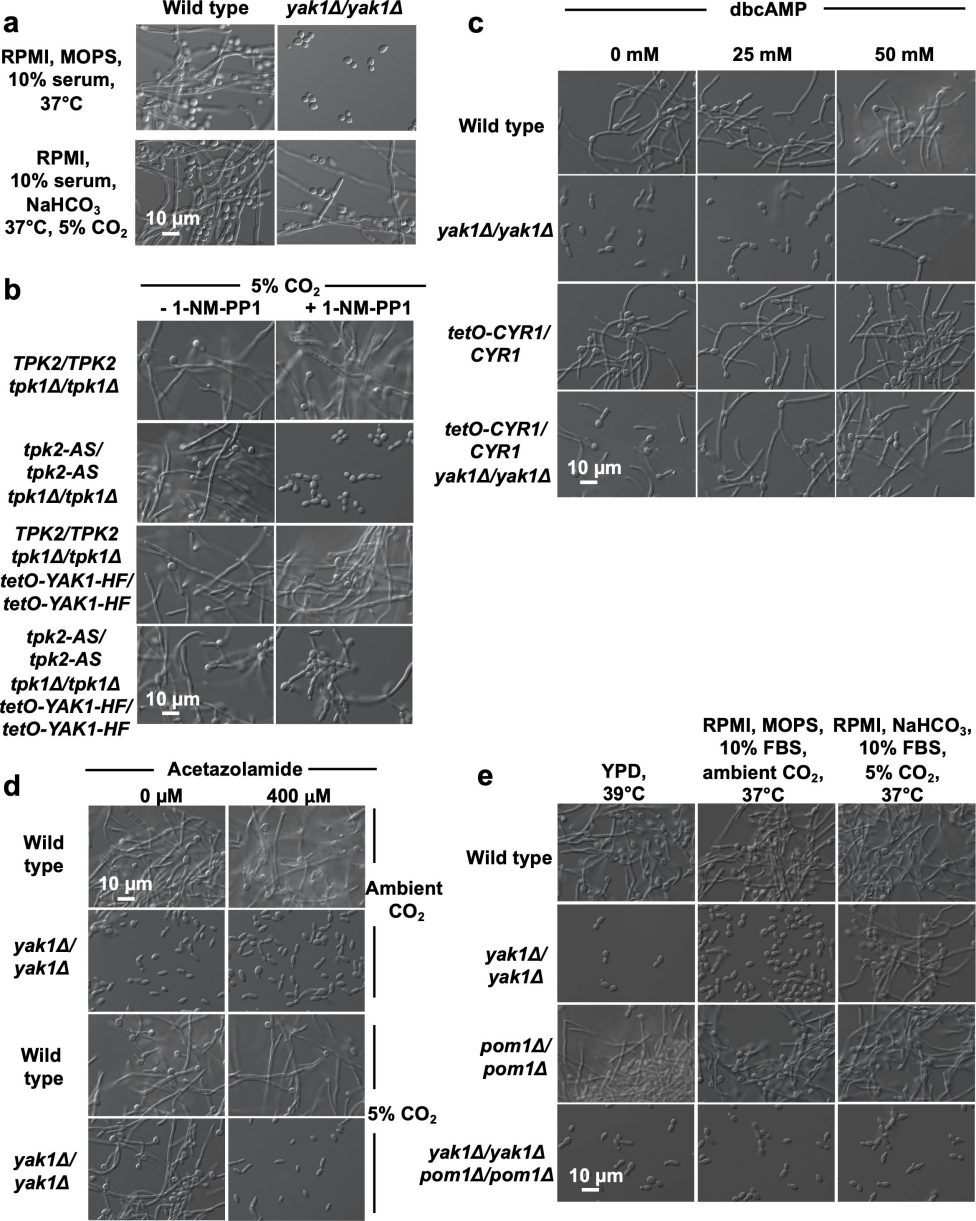
Yak1 regulates *C. albicans* morphogenesis in an environmentally-contingent manner. (a) Yak1 is not required for filamentation under 5% CO_2_. Wildtype or a *yak1* homozygous-deletion mutant were grown for 6 hours in RPMI buffered with either MOPS or NaHCO_3_ under either ambient or 5% CO_2,_ respectively. Data are representative of two biological replicates. (b) *YAK1* was overexpressed in the *TPK2* analog-sensitive strain (*TPK2-AS/TPK2-AS tpk1Δ/tpk1Δ*). In the presence of 1-NM-PP1 (2.5 µM), where PKA is inhibited, overexpression of *YAK1* rescued the defect in filamentation. Strains were grown at 37°C for 3 hours in RPMI buffered with NaHCO_3_ under 5% CO_2_. Data are representative of two biological replicates. (c) Under ambient (room air) CO_2_ concentration, overexpression of *CYR1* partially rescues the defect in filamentation of a *yak1-*deletion strain. Addition of exogenous dbcAMP, which directly stimulates PKA, further rescues this defect to wild-type levels of filamentation. Strains were grown at 37°C for 3 hours in RPMI buffered with MOPS under ambient CO_2_. Data are representative of two biological replicates. (d) Treatment with the carbonic anhydrase inhibitor, acetazolamide, restores the requirement for Yak1 for cells to filament under 5% CO_2_. Strains were grown at 37°C for 3 hours in RPMI buffered with either MOPS under ambient CO_2_ or NaHCO_3_ under 5% CO_2_, as indicated. Data are representative of two biological replicates. (e) Homozygous deletion of the predicted DYRK-encoding gene *POM1* in combination with homozygous deletion of *YAK1* blocks filamentation under both ambient and elevated CO_2_. Cells were grown in either YPD (42°C) or RPMI buffered with either MOPS under ambient CO_2,_ or NaHCO_3_ under 5% CO_2,_ at 37°C for 4 hours, as indicated. Light microscopy images are representative of two biological replicates.

While initial assessment of the *yak1* homozygous deletion mutant found the kinase to be an important mediator of filamentation under diverse conditions (including 37°C, RPMI, MOPS, 10% serum), we serendipitously discovered that both a *yak1* homozygous-deletion mutant and a Yak1^Y588F^ mutant were capable of filamenting under animal host-relevant concentrations of CO_2_ (37°C, RPMI, NaHCO_3_, 10% serum, 5% CO_2_) (Fig. 5a; Fig. S5a). This finding was unexpected as compromise of PKA activity blocks filamentation under both ambient and elevated CO_2_ concentrations (Fig. 5b). To test whether Yak1 continues to signal downstream of or parallel to PKA under physiological 5% CO_2_, we overexpressed *YAK1* in the analog-sensitive PKA mutant. Overexpression of *YAK1* rescued the filamentation defect observed in the presence of 1-NM-PP1 in our *TPK2* analog-sensitive strain, indicating that Yak1 still acts downstream of PKA or that its overexpression is sufficient to drive filamentation in the absence of PKA under elevated CO_2_ (Fig. 5b).

One explanation for this observation would be that physiological concentrations of CO_2_ hyperactivate the PKA pathway such that the additional stimulation bypasses the requirement for Yak1. Previous work found that bicarbonate directly stimulates Cyr1, the adenylate cyclase that acts upstream of PKA to regulate *C. albicans* morphogenesis (44–46). Based on the hypothesis that Yak1 is no longer required under elevated CO_2_ due to hyperactivation of the Ras1/cAMP/PKA pathway, we investigated whether hyperactivation of PKA could rescue filamentation in a *yak1* mutant under ambient CO_2_. First, we overexpressed *CYR1* using the *tetO* promoter in a strain of *C. albicans* with both alleles of *YAK1* deleted. Overexpression of *CYR1* partially rescued the defect observed in the *yak1* homozygous-deletion mutant under ambient CO_2_, with cells forming short, polarized projections. Next, we treated strains with the cell-permeable cAMP analog dibutyryl-cAMP (dbcAMP), which directly stimulates PKA activity to induce filamentous growth (47). Treatment with dbcAMP (50 mM) partially restored filamentation in a *yak1* homozygous-deletion mutant and fully restored filamentation in the *tetO-CYR1/CYR1 yak1Δ/yak1Δ* strain (Fig. 5c). This result strongly supports the hypothesis that hyperactivation of the Ras1/cAMP/PKA pathway bypasses the requirement for Yak1 seen under ambient CO_2_ conditions.

The carbonic anhydrase Nce103 catalyzes the conversion of CO_2_ to bicarbonate, which activates PKA signaling (44, 48). Using two carbonic anhydrase inhibitors, acetazolamide and methazolamide, we tested whether inhibition of Nce103 was capable of dampening PKA activity under 5% CO_2_ and restoring the requirement of Yak1 for hyphal morphogenesis under these conditions. In support of our model, treatment of a *yak1* homozygous-deletion mutant with either inhibitor blocked filamentation under physiological concentrations of CO_2_ (Fig. 5d; Fig. S5b). Overall, we conclude that hyperactivation of PKA is sufficient to bypass the requirement for Yak1 in supporting *C. albicans* morphogenesis.

### Orf19.5253 encodes a putative DYRK that enables filamentation under 5% CO_2_ in the absence of Yak1

**Fig 6:**
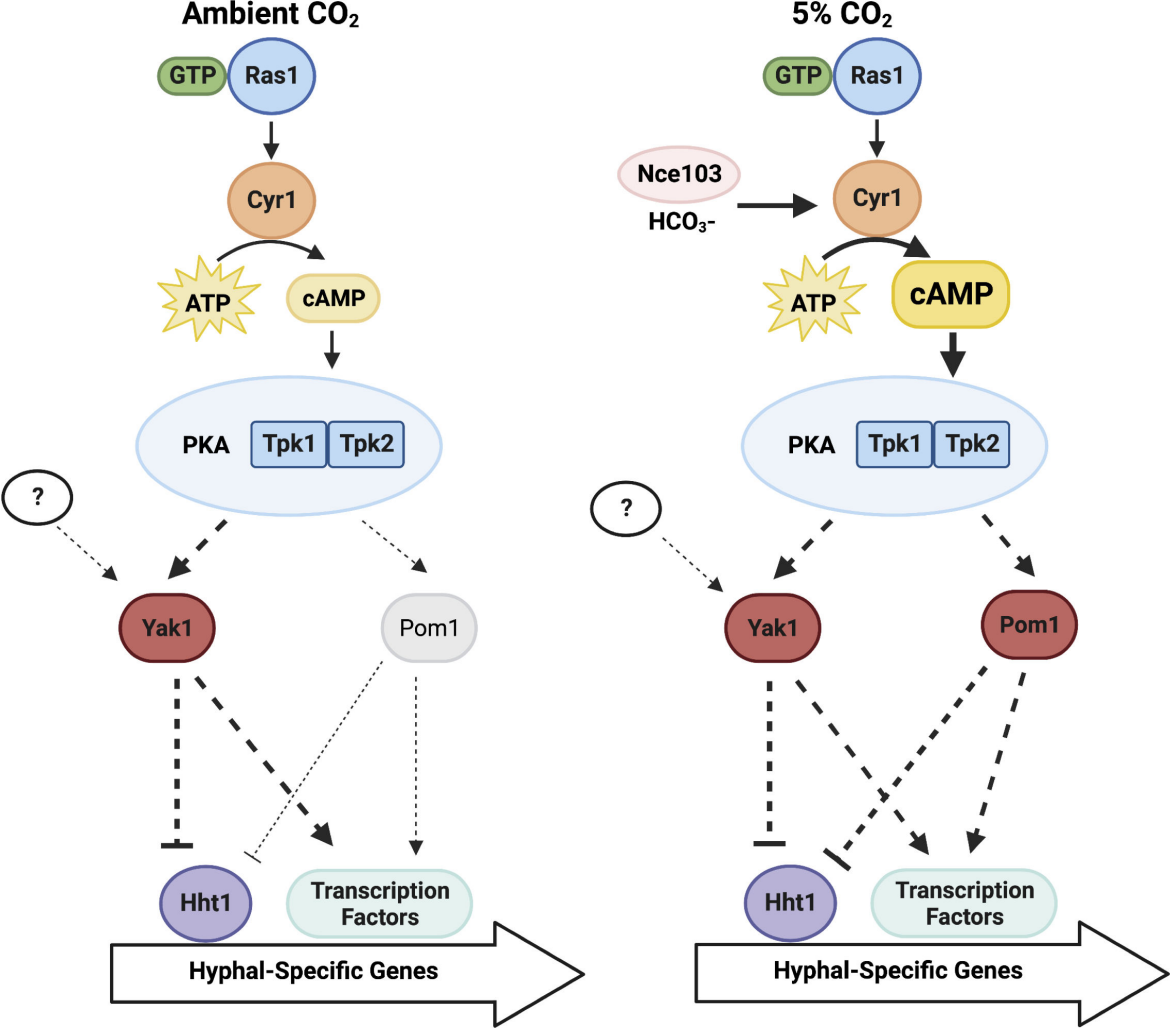
Model for Yak1 regulation of *C. albicans* morphogenesis in an environmentally-contingent manner. Under ambient CO_2_, Yak1 is required for *C. albicans* morphogenesis, where it acts downstream or parallel to PKA and it is dependent on core filamentation-regulatory transcription factors and histone H3 variant, Hht1. Under physiological concentrations of CO_2_, Yak1 acts downstream or parallel to PKA but is dispensable for *C. albicans* hyphal morphogenesis due to hyperactivation of the Ras1/cAMP/PKA pathway. A distinct DYRK, Pom1, exhibits a synthetic interaction with Yak1, such that deletion of both kinases results in a block in *C. albicans* morphogenesis under elevated concentrations of CO_2_. Figure made using Biorender.com.

Upon discovery that hyperactivation of PKA signaling could bypass a requirement for Yak1 during induction of hyphal morphogenesis, we hypothesized that there may be another cellular factor(s) that functions downstream or parallel to PKA to activate the filamentation program. Many fungal species, including *Aspergillus* spp*., Cryptococcus* spp., and the fission yeast *Schizosaccharomyces pombe*, encode two DYRKs, Yak1 and Pom1 (49). Surveying the *C. albicans* genome unveiled an uncharacterized gene, *orf19.5253,* which shares orthology to *S. pombe* and *Aspergillus nidulans* Pom1 (50, 51). Here, we will refer to *orf19.5253* in *C. albicans* as *POM1*. We hypothesized that this second predicted DYRK may act downstream or parallel to PKA to regulate filamentous growth under elevated CO_2_, even when Yak1 function is compromised. To test this prediction, we generated a homozygous *pom1*-deletion mutant in both wild-type and *yak1Δ/yak1Δ* genetic backgrounds. Although homozygous deletion of *POM1* did not result in a defect in hyphal morphogenesis induced by elevated temperature when cells were grown in either YPD, RPMI under ambient CO_2_, or RPMI under elevated CO_2_, deletion of both *POM1* and *YAK1* blocked filamentation under elevated levels of CO_2_ (Fig. 5e). Therefore, we propose a model in which, under 5% CO_2_, Pom1 can rescue the filamentation defect caused by *yak1* homozygous deletion (Fig. 6). Whether it might act upon Hht1 to alter chromatin structure as suggested for Yak1 (Fig. 4e) will require further investigation in future work.

### Yak1 is required for virulence in a mouse model of *C. albicans* dermatitis

**Fig 7:**
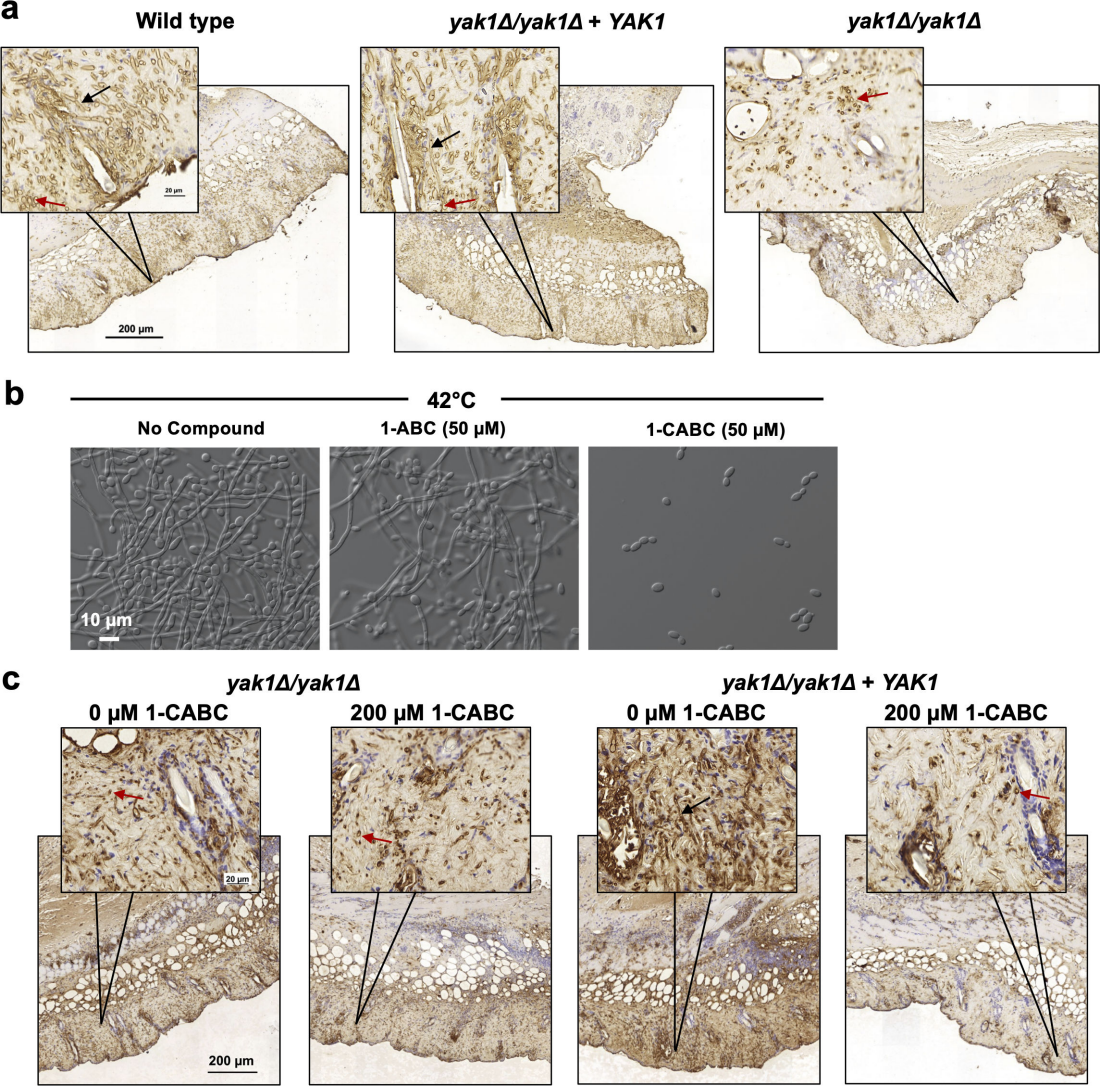
Yak1 is required for invasive filamentation in a mouse model of *C. albicans* dermatitis. (a) The dorsal skin of neutropenic BALB/c mice was infected with either wild-type (SN95), *yak1Δ/yak1Δ + YAK1*, or *yak1Δ/yak1Δ* strains by topical application of 1 × 10^7^ cells suspended in PBS. At 72-hours post-infection, mice were euthanized, and full thickness punch biopsies of infected skin were collected. Formalin-fixed, paraffin-embedded tissue sections were stained with anti-*C*. *albicans* antibody to assess morphology and extent of tissue invasion (brown signal). Black arrows indicate hyphal *C. albicans* cells and red arrows indicate *C. albicans* persisting as yeast cells. Inset images provide a view of the indicated tissue regions at higher magnification to visualize *C. albicans* morphology. Images are representative of two tissue sections prepared for each of the three animals infected in the study. (b) 1-carboxylic acid beta-carboline (1-CABC) is more potent than 1-ABC in blocking filamentation. Wild-type *C. albicans* was grown in YPD at 42°C in the absence or presence of 1-ABC or 1-CABC for 6 hours to induce filamentation. Light microscopy images are representative of three biological replicates. (c) Neutropenic BALB/c mice were infected by topical application of either *yak1Δ/Δ* or *yak1Δ/Δ + YAK1 C. albicans* (1 × 10^7^ cells/mL) prepared in PBS with or without 42.4 µg/mL solution of 1-CABC. Topical treatment with 1-CABC or PBS was repeated daily until 72-hours post-infection when mice were euthanized, and punch biopsies were collected. Formalin-fixed, paraffin-embedded tissue sections were stained with an anti-*C*. *albicans* antibody to assess morphology and extent of tissue invasion (brown signal). Black arrows indicate hyphal *C. albicans* and red arrows indicate *C. albicans* persisting in yeast morphology. Inset images provide a view of the indicated tissue regions at higher magnification to visualize *C. albicans* morphology. Images are representative of two tissue sections prepared for each of the five animals infected in the study.

Previous work reported that Yak1 was not required for virulence in a systemic or oral model of candidiasis (23). Here, we found that Yak1 is dispensable for filamentous growth under physiological concentrations of CO_2_, likely explaining the wild-type virulence phenotype a *yak1* mutant displays in the mouse models tested. However, *C. albicans* is also a frequent colonizer of the skin and often causes superficial infection (fungal dermatitis) in susceptible hosts. These common infections are associated with significant morbidity in their own right but can also progress to systemic disease in severely immunocompromised individuals (52). To evaluate the virulence of a *yak1* homozygous-deletion mutant under ambient atmospheric CO_2_ levels, we turned to a standard fungal dermatitis model in neutropenic (cyclophosphamide-preconditioned) mice. Fungal inoculum containing either a parental wild-type strain, a *yak1* homozygous-deletion strain, or the deletion strain complemented with one copy of *YAK1* was applied to denuded skin. Three days post-infection, full thickness punch biopsies of the infected area were collected and evaluated for fungal burden and morphology. Fungus was identified using an anti-*C*. *albicans* antibody. In this model, the wild-type and complemented strains caused robust infection with many polarized cells seen invading the dermis (Fig. 7a, black arrows). In contrast, infection with the *yak1* homozygous-deletion mutant resulted in detection of fewer fungi that appeared to be blocked in morphogenesis as yeast cells (Fig. 7a, red arrows).

Encouraged by the results using genetically-modified strains, we investigated the potential of pharmacologically targeting Yak1 as a strategy to combat fungal dermatitis. In previous work, we identified a Yak1 inhibitor secreted by *Lactobacillus* spp., 1-ABC (22). To identify analogs of 1-ABC with greater potency, we tested a panel of beta-carbolines for their ability to block *C. albicans* filamentation. A close structural analog, 1-carboxylic acid beta-carboline (1-CABC), was identified with improved potency (Fig. 7b). To determine whether 1-CABC could block filamentation and ameliorate infection in a neutropenic mouse model of fungal dermatitis, we treated mice infected with the complemented strain with 1-CABC applied topically. This treatment phenocopied infection with a *yak1* homozygous-deletion strain, blocking the formation of filamentous cells (Fig. 7c) and reducing the extent of disease with no evident toxicity to the involved skin. Thus, with further development, pharmacological inhibition of Yak1 with a beta-carboline may prove useful in the treatment of dermatitis caused by *C. albicans*.

## DISCUSSION

The opportunistic fungal pathogen *C. albicans* has evolved a complex repertoire of mechanisms to adapt to diverse environmental conditions and to thrive within a human host. Specifically, its ability to transition between yeast and filamentous morphologies upon exposure to host-relevant cues plays important roles in its pathogenicity. Here, we demonstrate that the DYRK Yak1 acts within the Ras1/cAMP/PKA pathway to regulate *C. albicans* morphogenesis in an environmentally-contingent manner. In *C. albicans,* Yak1 functions downstream or parallel to PKA and is dependent on core transcription factors to regulate the yeast-to-hyphae transition in response to a broad range of distinct stimuli under ambient CO_2_. Interestingly, hyperactivation of Ras1/cAMP/PKA signaling by host-relevant concentrations of CO_2_ bypasses the requirement for Yak1 for filamentous growth. To identify the factor(s) downstream or parallel to PKA, which mediates filamentation under elevated CO_2_, we identified and characterized another DYRK encoded in the *C. albican*s genome, Pom1, where homozygous deletion of both *POM1* and *YAK1* blocks filamentation under elevated CO_2_ (Fig. 6). Finally, we demonstrated that Yak1 is required for hyphal morphogenesis in a mouse model of *C. albicans* dermatitis and that pharmacological inhibition of Yak1 with 1-CABC impaired filamentous invasion of the dermis in this model. Overall, this work provides actionable mechanistic insights into the role DYRKs play in regulating a core virulence trait in *C. albicans*.

Yak1 is a member of the evolutionarily-conserved family of Ser/Thr DYRKs, a class of kinases characterized by a YXY motif in the activation loop of their catalytic domain. Despite this conservation at the structural level, we describe evolutionary divergence in the regulation of Yak1 between the model yeast *S. cerevisiae* and the pathogen *C. albicans*. Previous work found that Yak1 acts downstream of PKA to regulate growth and stress responses in *S. cerevisiae,* where PKA negatively regulates Yak1 kinase activity (53). Here, we demonstrate that Yak1 likely also acts downstream of PKA in *C. albicans*, but the regulation appears to be positive in nature, where the loss of either PKA or Yak1 blocks *C. albicans* hyphal morphogenesis. Paradoxically, substitutions of five Ser/Thr residues predicted to be phosphorylated by PKA to phospho-ablation sites resulted in constitutive filamentation under basal conditions.

Several potential explanations for the disparity observed remain to be explored. These include, but are not limited to, PKA directly phosphorylating alternate residues in Yak1 to positively regulate its function or mutation to alanine of all five sites causing a conformational change in the protein, which leads to its constitutive activation. Alternatively, PKA could indirectly regulate Yak1 activity by phosphorylating other kinases or phosphatases that then act upon Yak1. Additionally, we found that the auto-phosphorylated tyrosine residue, Y588, is required for Yak1 activity in *C. albicans*, but unlike the situation reported in other organisms, its phosphorylation status does not markedly alter the stability of the protein (31, 32). Perhaps phospho-ablation of the five residues we examined results in increased autophosphorylation of Y588 and constitutive rather than regulated functioning of the kinase.

After tyrosine autophosphorylation occurs, DYRKs act as strict Ser/Thr kinases for exogenous substrates. In *S. cerevisiae,* several substrates of Yak1 have been identified, including Bcy1, a regulatory subunit of PKA (54), and the stress-responsive transcription factors, Hsf1 and Msn2 (30). Interestingly, in *C. albicans* the heat shock-regulated transcription factor Hsf1 regulates filamentous growth, such that both overexpression and depletion of *HSF1* induce morphogenesis in the absence of external cues (55). While it remains unknown whether *C. albicans* Yak1 regulates Hsf1 function, it would certainly be an interesting avenue for future investigation. Regardless, our work complements previous studies that have found significant divergence in the regulation of signal transduction pathways between the two species (15, 56, 57), as well as research that describes substantial morphogenetic reprogramming between the model yeast and fungal pathogen (15, 58). Therefore, to improve our understanding of the *C. albicans* yeast-to-filament transition, it will be imperative to perform further studies using the pathogen itself rather than a surrogate model.

We found that overexpression of many transcription factors that positively regulate *C. albicans* morphogenesis can bypass the defect observed in a *yak1* homozygous-deletion mutant. This led us to investigate whether Yak1 may impact access to DNA to enable the filamentation program. Consistent with this hypothesis, deletion of the gene encoding histone H3 variant, Hht1, rescued the filamentation defect observed in a *yak1* homozygous deletion-mutant, suggesting Yak1 may regulate chromatin remodeling in *C. albicans*. Consistent with this observation, studies in *S. cerevisiae* found that Yak1 diminishes the nuclear localization of Msi1, as well as its association with Cac1 in the chromatin assembly factor I complex, suggesting a possible role for Yak1 in regulating chromatin structure (59). Further studies will be required to elucidate if and how *C. albicans* Yak1 affects chromatin dynamics and the implications this might have for regulating specific and/or global gene expression.

Here, we also demonstrate that Yak1 orchestrates *C. albicans* morphogenesis in an environmentally-contingent manner. Although Yak1 was required for filamentation across diverse inducing cues, including 10% serum, nutrient limitation, and elevated temperature, the kinase was not required under physiological concentrations of CO_2_ found in mammals. This provides a possible explanation as to why, prior to this study, Yak1 inhibition only demonstrated efficacy in biofilm models of disease performed under room air, and why Yak1 was not required for virulence in a systemic or oral model of *C. albicans* morphogenesis (23). Investigating further, we found that Yak1 is dispensable for filamentation under 5% CO_2_ due to hyperactivation of the PKA pathway by the bicarbonate ion (46). Extending from this result, we described a new role for the putative DYRK, Pom1, as a regulator of morphogenesis that can substitute for Yak1 under physiological CO_2_. In *A. nidulans,* the *POM1* ortholog, *pomA* was identified as a negative regulator of cytokinesis via signaling through HogA and SepH (60). Similarly, in *S. pombe*, Pom1 activation prevents septation at cell tips at least in part through phosphorylation of the F-BAR protein Cdc15 (61). Notably, impairment of cell cycle progression in *C. albicans* leads to polarized growth in the absence of additional inducing cues (62). Further work will be needed to learn how Pom1 regulates *C. albicans* filamentation as well as to evaluate whether genetic impairment of both *YAK1* and *POM1* would result in a virulence defect in systemic models of *C. albicans* infection that involves exposure of the organism to physiological CO_2_ levels.

As the frequency and severity of fungal infections continue to escalate, the discovery and development of novel therapeutic interventions to combat drug-resistant diseases are of great urgency. The need for new agents applies to both systemic fungal infections as well as superficial diseases such as vaginal candidiasis and dermatitis, which afflict millions of individuals annually. Here, we demonstrate that Yak1 is required for hyphal morphogenesis under ambient CO_2_ in a model of *C. albicans* dermatitis. We also find that the novel Yak1 inhibitor, 1-CABC, can phenocopy the filamentation defect observed during infection by a *C. albicans yak1* homozygous-deletion mutant. This finding provides a rationale to support the pursuit of Yak1 inhibition by beta-carbolines as a promising therapeutic strategy to combat *C. albicans* dermatitis. Ongoing work by others is evaluating DYRK inhibitors for the treatment of human diseases, such as Down syndrome, demonstrating that systemic exposure is well tolerated (63, 64). These investigations coupled with advanced structure-activity-relationship studies may be able to facilitate the identification of compounds with activity against both Yak1 and Pom1, potentially broadening the therapeutic potential of DYRK inhibitors to the treatment of systemic infections caused by *C. albicans* and perhaps other fungi, which require DYRK-like activity for virulence. Thus, as demonstrated here, deciphering the circuitry governing *C. albicans* filamentation under host-relevant environmental conditions has the potential to not only provide biological insights into the signaling pathways that govern this important virulence trait but also reveal new strategies for crippling fungal pathogens.

## MATERIALS AND METHODS

### Growth conditions

All strains used in this study are listed in Table S1, all plasmids used for strain construction are listed in Table S2, and all oligonucleotide sequences used in this study are listed in Table S3. Strain construction details are described in Methods in the supplemental material. All *C. albicans* strains used in this study were stored at −80°C in rich medium (YPD) supplemented with 25% glycerol by volume. YPD was prepared with 1% yeast extract, 2% glucose, and 2% bactopeptone. Unless otherwise indicated, *C. albicans* strains were grown in standard conditions in YPD at 30°C. Where indicated, strains were grown in the presence of DOX dissolved in filter-sterilized water (Doxycycline Hydrochloride, BioBasic, DB0889). The beta-carbolines used in this study were 1-ABC (MolPort-039-338-922) and 1-CABC (Enamine # EN300-205447), prepared as 20 mM dimethyl sulfoxide (DMSO) solutions and stored at −20°C. The bulky ATP analog, 1-NM-PP1 (Cedarlane #B1299-10) was prepared as a 20 mM stock in DMSO and stored at −20°C.

### *C. albicans* filamentation assay

*C. albicans* hyphal morphogenesis was induced by sub-culturing strains from an overnight culture grown at 30°C under shaking conditions. Saturated cells were sub-cultured to an OD_600_ of 0.01 in the relevant inducing cue, as described. The cues tested were YPD containing 10% (vol/vol) heat-inactivated newborn bovine calf serum (NBCS, Gibco #26010066) for 3 hours at 37°C, 5 mM N-acetylglucosamine (GlcNAc) in YPD at 37°C for 3 hours, Spider medium at 37°C for 6 hours, or RPMI medium with 10% (vol/vol) NBCS and buffered with either 3-morpholinopropane-1-sulfonic acid (MOPS) or NaHCO_3_ at 37°C for 4 hours. Media for each cue were prepared as previously described (65). For all microscopy experiments, cells were viewed either using differential interference contrast microscopy or using fluorescence microscopy with an enhanced green fluorescent protein, or DAPI channels on a Zeiss Axio Imager M1 microscopy (Carl Zeiss) at the same exposure time. A minimum of three independent fields of view were captured for each condition. All images are representative of three technical replicates and two biological replicates.

### *C. albicans* glycogen accumulation

*C. albicans* strains were grown overnight in YPD at 30°C under shaking conditions. The next day, 1 mL of each culture was washed twice with sterile PBS and diluted to an OD_600_ = 1. A total of 5 µL of each strain was spotted onto YPD agar either with or without 2.5 µM 1-NM-PP1. Plates were left to grow at 30°C for 24 hours. The lid of a Petri dish was then filled with iodine crystals and the plates were inverted over the bed of crystals for 5 minutes to stain for glycogen. Stained plates were then imaged on a ChemiDoc system.

### Quantitative reverse transcription-PCR

All cells were grown overnight in YPD at 30°C under shaking conditions. The following day, cells were sub-cultured into either YPD or YPD plus 20 µg/mL DOX and grown overnight again at 30°C under shaking conditions. The next day, cells were diluted to an OD_600_ of 0.1 in YPD with or without 20 µg/mL DOX and grown at 30°C to mid-log phase (about 3–4 hours). Cultures were flash-frozen and stored at −80°C overnight. RNA extraction, complementary DNA synthesis, and qPCR were performed as previously described (62). Briefly, qPCR was performed using the Fast SYBR green master mix (Applied Biosystems) and a Bio-Rad CFX384 real-time system using the following cycling conditions: 95°C for 3 minutes, followed by 40 cycles of 95°C for 10 seconds and 60°C for 30 seconds. All PCR reactions were performed in technical quadruplicate for two biological replicates. The resulting data were analyzed using the Bio-Rad CFX Manager 3.1 software. Data are normalized to the *ACT1* reference gene for *C. albicans*. All error bars represent the standard error of the mean of technical quadruplicates.

### Western blotting

For all western blots, cells were first grown overnight at 30°C under shaking conditions. The following day, cells were diluted to an OD_600_ of 0.1 in 40 mL YPD and grown at 30°C to mid-log phase (about 3–4 hours). Where indicated, cells were sub-cultured into YPD supplemented with 2.5 µM 1-NM-PP1. Cells were then flash frozen and stored at −80°C. For all samples probing for Yak1 protein levels, protein was extracted under either non-denaturing or denaturing conditions, as indicated. For samples probing Efg1 and Flo8 protein levels, protein was extracted under denaturing conditions. For the denaturing protein extraction, cell pellets were resuspended in 650 µL of 427 mM NaOH and 1.73% beta-mercaptoethanol (vol/vol). Samples were incubated on ice for 5 minutes, and then 150 µL of 50% trichloroacetic acid was added. Following an additional 5-minute incubation, protein was collected by centrifugation at 14,000 × *g* for 7 minutes at 4°C. The supernatant was removed, and the pellet was resuspended in 150 µL of loading buffer [40 mM Tris-HCl pH 6.8, 5% (wt/vol) SDS, 100 mM NaEDTA, 0.1 mg/mL bromophenol blue, 0.5 g/mL urea, 0.01% beta-mercaptoethanol] and incubated at 42°C for 10 minutes. Debris was collected and removed by centrifugation at 12,000 × *g* for 2 minutes. Samples were then loaded on an SDS-PAGE gel, as indicated.

For non-denaturing protein extraction, cells were resuspended in lysis buffer [20 mM Tris pH 7.5, 100 mM KCl, and 5 mM MgCl_2_, supplemented with cOmplete Roche Protease inhibitor tabs (Roche #11836170001) and Roche PhosSTOP phosphatase inhibitor tabs (Roche #4906845001)]. Samples were lysed by bead beating twice for 3 minutes each, with 4 minutes on ice in between. Next, the sample was sonicated at 30% amplitude for 20 seconds, three times, with samples stored on ice in between sonication (Misonix S-4000 dual horn with 3/4-inch probes). Magnesium (II) chloride and IGEPAL-CA630 were then added to the sample to a final concentration of 2 mM and 0.02%, respectively. The supernatant was collected and spun for 15 minutes at 14,000 × *g* at 4°C. The supernatant was collected, and the samples were spun again at 14,000 × *g* at 4°C for 10 minutes. Protein was quantified using the DC Protein Assay Kit II (Bio-Rad #5000112). For each sample, 15 µg was loaded into an SDS-PAGE gel.

For all western blot analyses, an anti-FLAG antibody was used to probe for epitope-tagged protein levels. Following the transfer of the SDS-PAGE gel to a nitrocellulose membrane (12 hours, 4°C, 100 mA), the membrane was blocked with 5% skim milk prepared in 1× tris-buffered saline-Tween 20 (TBS-T) for 1 hour. The membrane was then probed with an anti-FLAG antibody prepared in 5% skim milk for 1 hour [mouse monoclonal Anti-FLAG M2-Peroxidase (HRP), Sigma-Aldrich #A8592]. Following three washes in 1× TBS-T for 10 minutes each, the blot was developed using the Clarity Western ECL Substrate Kit (Bio-Rad, #1705060), and the blot was imaged on a ChemiDoc Bio-Rad imager.

### Mouse model of *C. albicans* dermatitis

BALB/c mice were rendered neutropenic by treatment with cyclophosphamide (150 mg/kg of body weight 4 days prior to infection and 100 mg/kg of body weight 1 day prior to infection). To prepare the skin for fungal infection, hair was removed on the dorsal surface using the depilatory agent Nair and then the surface was tape stripped seven times to remove the outermost layer of skin (66). Mice were then challenged with 1 × 10^7^ cells in 100 µL of either *yak1Δ/yak1Δ + YAK1* or a *yak1Δ/yak1Δ* strain of *C. albicans*. In some experiments, infected skin was treated with either PBS vehicle control or 1-CABC (200 µM; 0.004%, wt/vol) prepared in PBS. Topical applications of 100 µL were performed at the time of fungal inoculation and again at 24- and 48-hours post-infection. Seventy-two hours after infection, mice were euthanized and skin tissue from the infected area was collected as a punch biopsy. The skin was fixed in formalin and then paraffin-ed. Tissue sections (4 µm) were stained with an anti-*C*. *albicans* antibody (1:1,000, Abcam ab53891) followed by incubation with an HRP Reagent solution pre-made in the ImmPRESS Horse Anti-Rabbit IgG Polymer Kit, Peroxidase-Biolynx (Vector Laboratories, MP-7401). Detection with diaminobenzadine was conducted per the manufacturer’s recommendations (ImmPACT DAB Substrate Kit, Peroxidase-Biolynx, Vector Laboratories, SK-4105).

## Data Availability

Source data are provided with this paper and all additional data, including raw data and images associated with all figures, is available upon reasonable request to L.E.C., the corresponding author.

## SUPPLEMENTAL MATERIAL

Fig. S1 mbio.02183-23-s0001.tiff Alignment of *C. albicans* and *S. cerevisiae* Yak1 amino acid sequences.(67)

Fig. S2 mbio.02183-23-s0002.tiff Genes under control of a *tetO* promoter are expressed at higher levels than wild type in the absence of doxycycline.

Fig. S3 mbio.02183-23-s0003.tiff Phospho-ablative (Ser/Thr>>Ala) or phospho-mimetic (Ser/Thr>>Asp) substitutions at all five predicted PKA phosphorylation sites in Yak1 does not alter expression of YAK1 mRNA.

Fig. S4 mbio.02183-23-s0004.tiff Deletion of YAK1 does not alter Flo8 localization.

Fig. S5 mbio.02183-23-s0005.tiff The role of Yak1 in regulating *C. albicans* morphogenesis is CO_2_ sensitive.

Tables S1-S3 and Supplemental Methods mbio.02183-23-s0006.pdf Strains, plasmids, and oligonucleotides used in this study along with strain construction details.

ASM does not own the copyrights to Supplemental Material that may be linked to, or accessed through, an article. The authors have granted ASM a non-exclusive, world-wide license to publish the Supplemental Material files. Please contact the corresponding author directly for reuse.
