# Supplementary figures and images for: DYRK-family kinases regulate Candida albicans morphogenesis and virulence through the Ras1/PKA pathway
Source: mBio. 2023 Nov 28;14(6):e02183-23. doi: 10.1128/mbio.02183-23 (PMC10746247; doi:10.1128/mbio.02183-23)

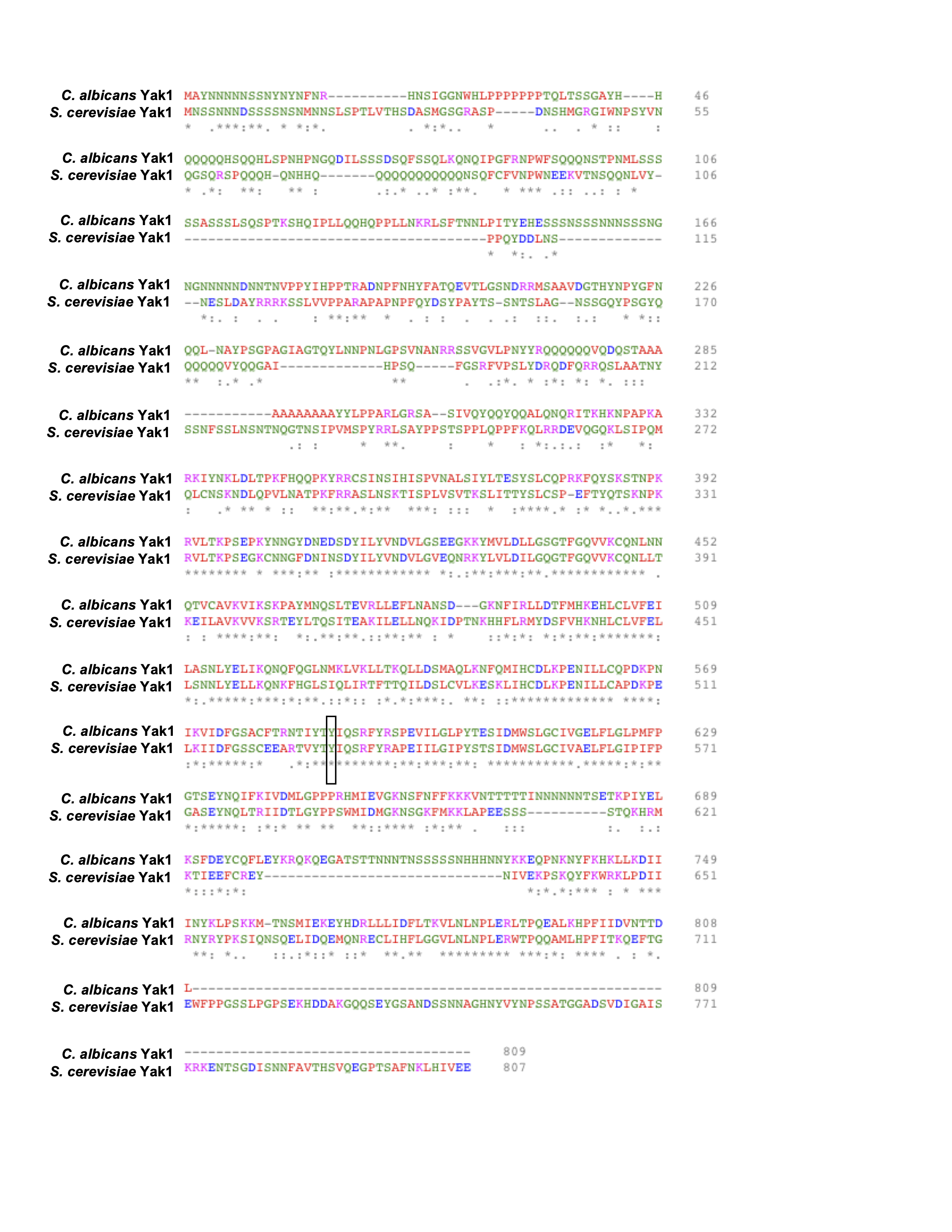

Supplement: Fig. S1 — Alignment of C. albicans and S. cerevisiae Yak1 amino acid sequences.(67) [file mbio.02183-23-s0001.tiff]

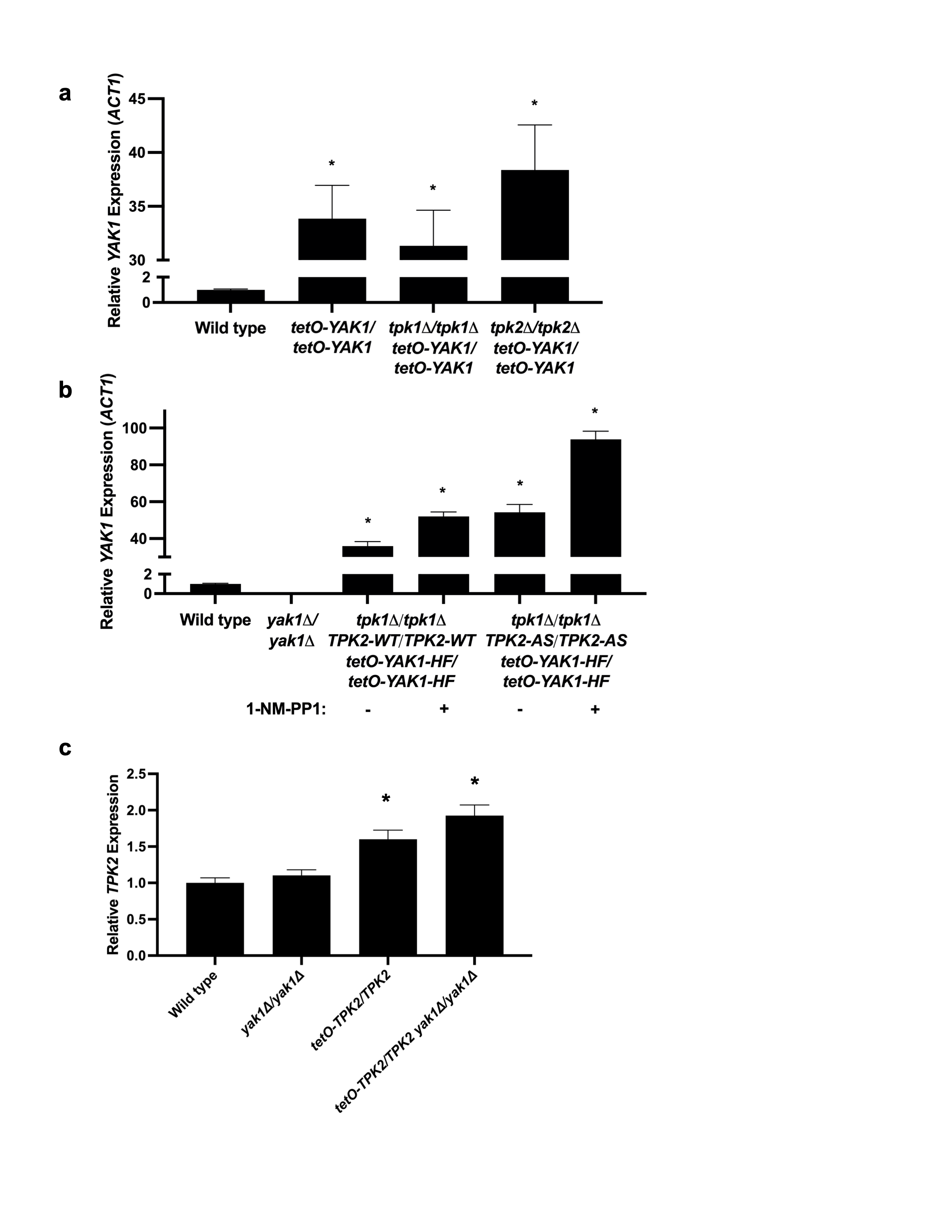

Supplement: Fig. S2 — Genes under control of a tetO promoter are expressed at higher levels than wild type in the absence of doxycycline. [file mbio.02183-23-s0002.tiff]

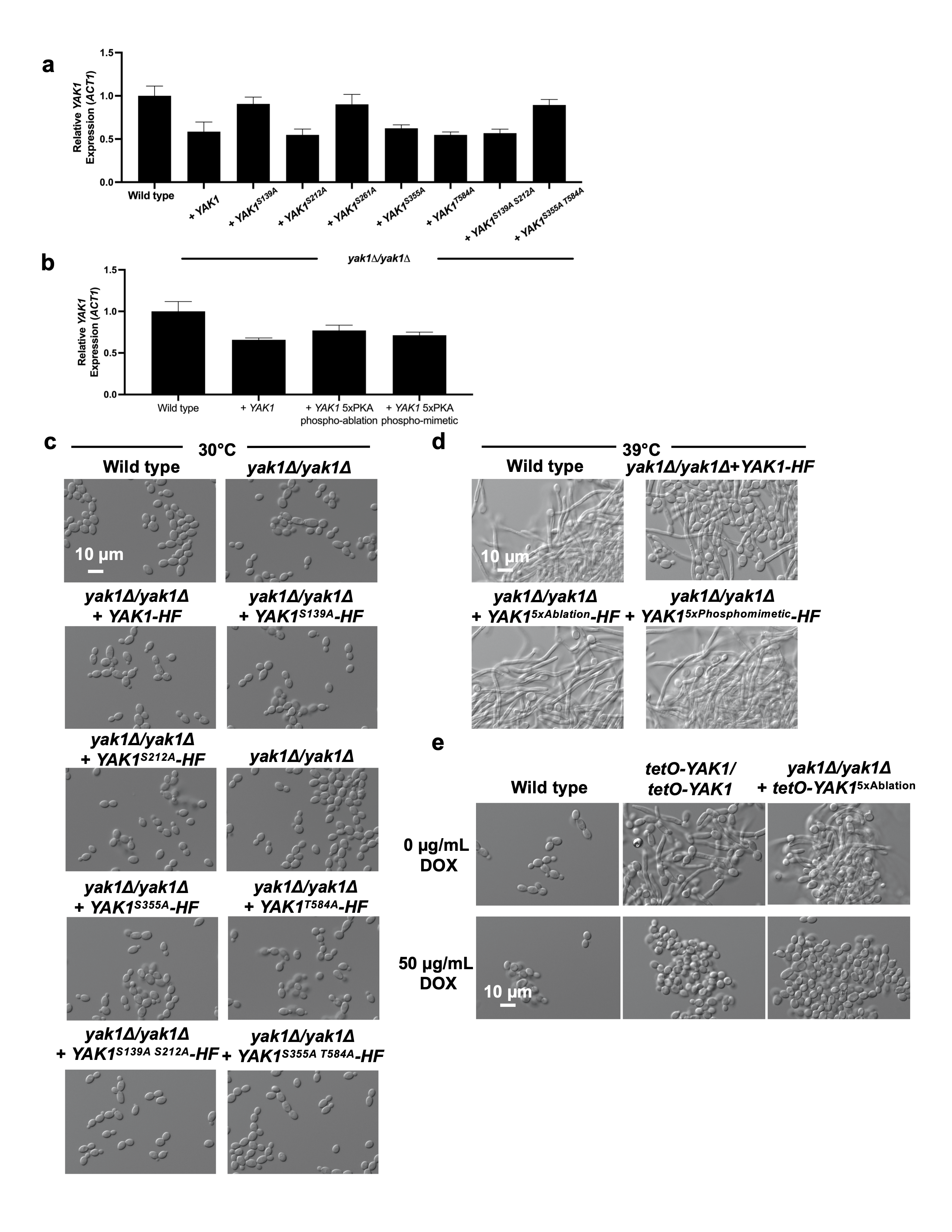

Supplement: Fig. S3 — Phospho-ablative (Ser/Thr>>Ala) or phospho-mimetic (Ser/Thr>>Asp) substitutions at all five predicted PKA phosphorylation sites in Yak1 does not alter expression of YAK1 mRNA. [file mbio.02183-23-s0003.tiff]

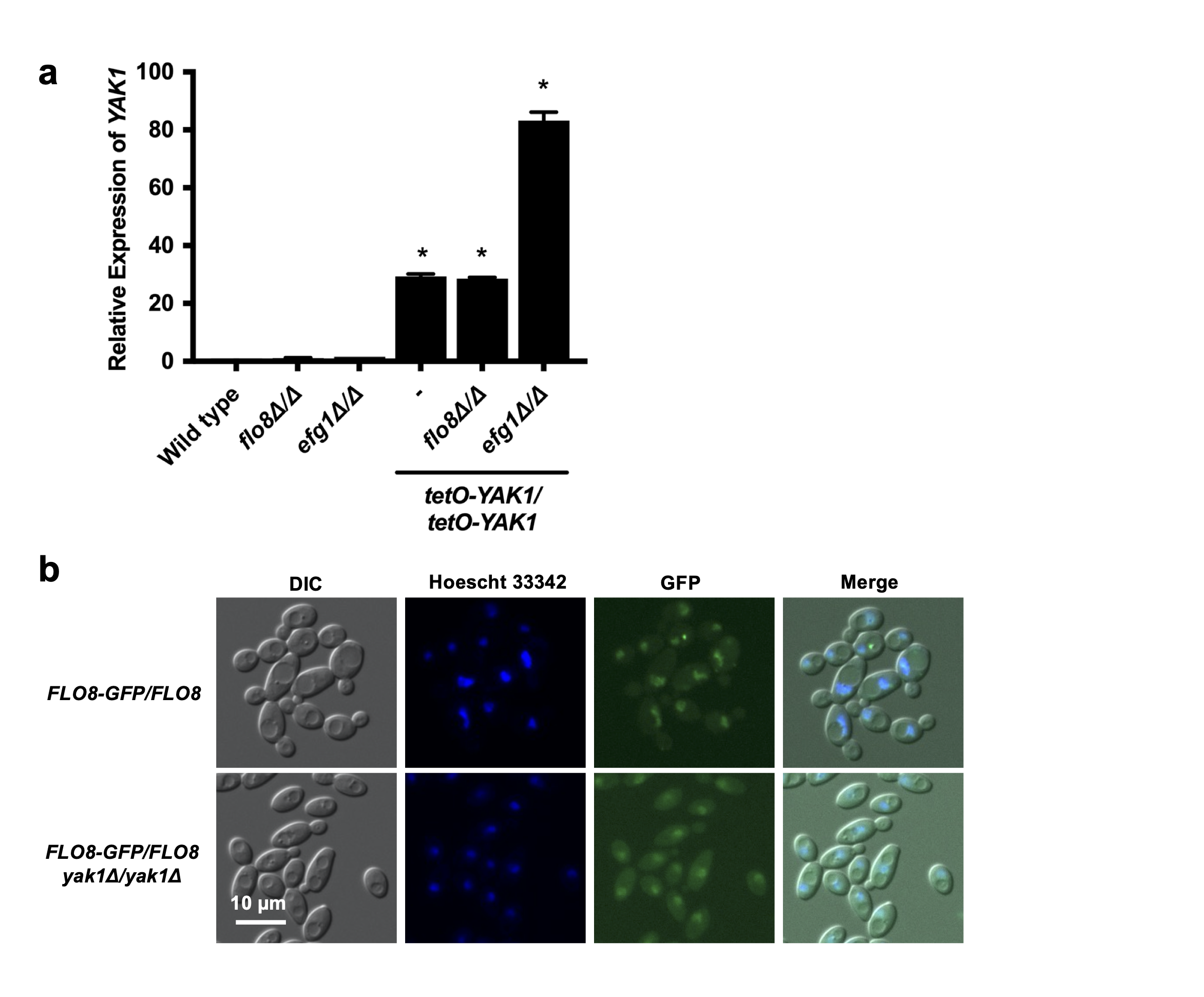

Supplement: Fig. S4 — Deletion of YAK1 does not alter Flo8 localization. [file mbio.02183-23-s0004.tiff]

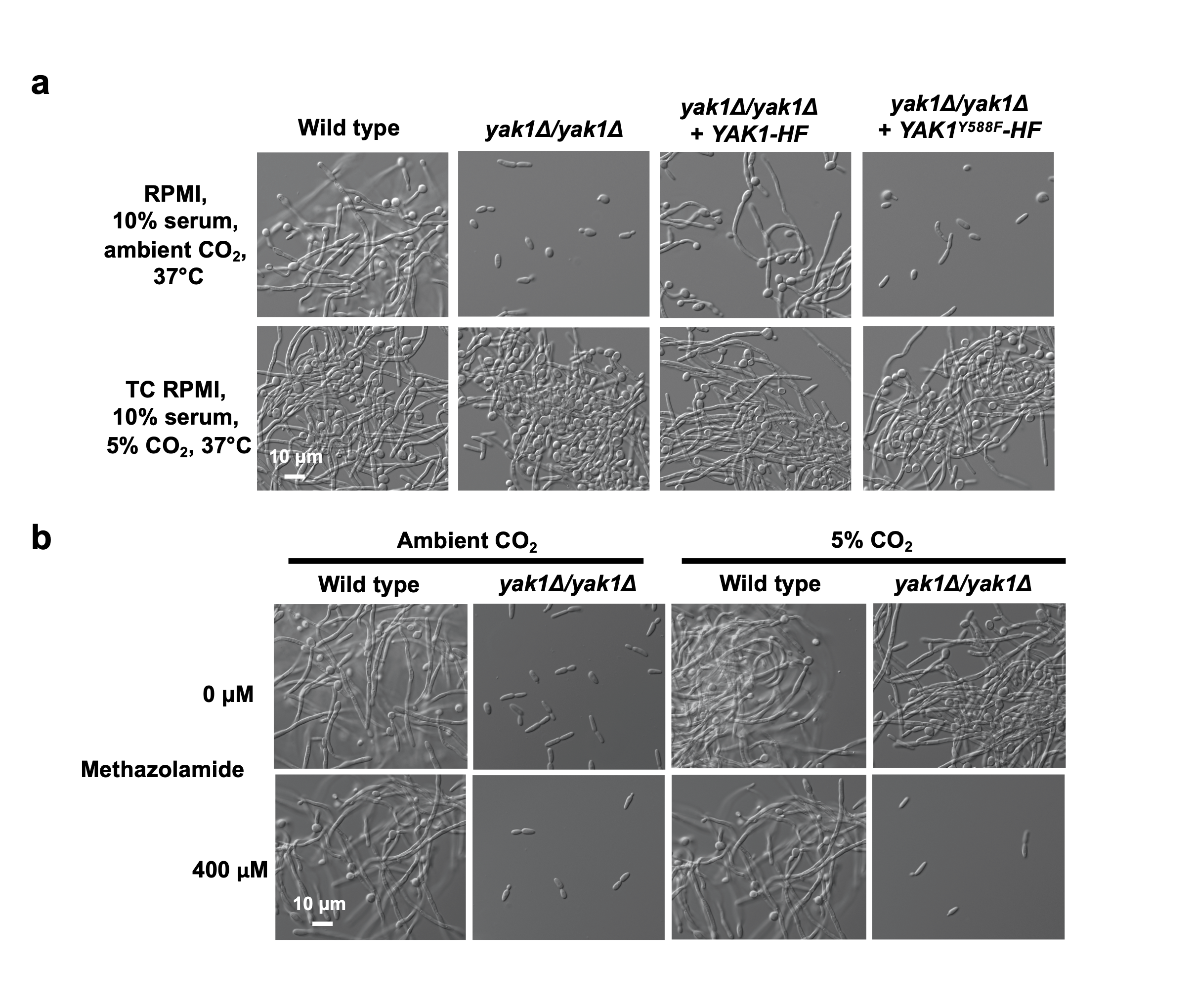

Supplement: Fig. S5 — The role of Yak1 in regulating C. albicans morphogenesis is CO2 sensitive. [file mbio.02183-23-s0005.tiff]
