# Supplementary material for: DYRK-family kinases regulate Candida albicans morphogenesis and virulence through the Ras1/PKA pathway
Source: mBio. 2023 Nov 28;14(6):e02183-23. doi: 10.1128/mbio.02183-23 (PMC10746247; doi:10.1128/mbio.02183-23)
Supplement: Tables S1-S3 and Supplemental Methods — Strains, plasmids, and oligonucleotides used in this study along with strain construction details. [file mbio.02183-23-s0006.pdf]

Supplementary Table 1: Strains used in this study.

Accession Number	Strain Name	Genotype	Source
CaLC155	SC5314	Prototrophic	[1]
CaLC239	SN95	*arg4Δ/arg4Δ his1Δ/his1Δ URA3/ura3::imm434 IRO1/iro1::imm434*	[2]
CaLC2897	*flo8Δ/flo8Δ*	*arg4Δ/arg4Δ his1Δ/his1Δ URA3/ura3::imm434 IRO1/iro1::imm434 flo8::FRT/flo8::FRT*	[3]
CaLC5654	*FLO8/FLO8*-GFP	*arg4Δ/arg4Δ his1Δ/his1Δ URA3/ura3::imm434 IRO1/iro1::imm434 FLO8/FLO8-GFP-SAT1*	[4]
CaLC6702	*tpk2Δ/tpk2Δ*	*his1Δ/his1Δ URA3/ura3::imm434 IRO1/iro1::imm434 tpk2::ARG/tpk2::ARG*	This study.
CaLC6699	*tpk1Δ/tpk1Δ*	*his1Δ/his1Δ URA3/ura3::imm434 IRO1/iro1::imm434 tpk1::ARG/tpk1::ARG*	This study.
CaLC7214	*yak1Δ/yak1Δ*	*his1Δ/his1Δ URA3/ura3::imm434 IRO1/iro1::imm434 yak1::ARG/yak1::ARG*	This study.
CaLC7435	*tetO-YAK1/tetO-YAK1*	*arg4Δ/arg4Δ his1Δ/his1Δ URA3/ura3::imm434 IRO1/iro1::imm434 HygB-TAR-tetO-YAK1/HygB-TAR-tetO-YAK1*	This study.
CaLC8144	*efg1Δ/efg1Δ*	*arg4Δ/arg4Δ his1Δ/his1Δ URA3/ura3::imm434 IRO1/iro1::imm434 efg1::HYG/efg1::HYG*	This study.
CaLC8188	*tetO-YAK1-GFP/*	*arg4Δ/arg4Δ his1Δ/his1Δ URA3/ura3::imm434*	This study.

	tetO-YAK1-GFP	*IRO1/iro1::imm434 HygB-TAR-tetO-YAK1-GFP-SAT1/HygB-TAR-tetO-YAK1-GFP-SAT1*	
CaLC8189	*flo8Δ/flo8Δ*<br><br>*tetO-YAK1/tetO-YAK1*	*arg4Δ/arg4Δ his1Δ/his1Δ URA3/ura3::imm434 IRO1/iro1::imm434 flo8::FRT/flo8::FRT SAT1-tetO-YAK1/SAT1-tetO-YAK1*	This study.
CaLC8190	*tpk2Δ/tpk2Δ*<br><br>*tetO-YAK1/tetO-YAK1*	*his1Δ/his1Δ URA3/ura3::imm434 IRO1/iro1::imm434 tpk2::ARG/tpk2::ARG SAT1-tetO-YAK1/SAT1-tetO-YAK1*	This study.
CaLC8297	*tpk1Δ/tpk1Δ*<br><br>*tetO-YAK1/tetO-YAK1*	*his1Δ/his1Δ URA3/ura3::imm434 IRO1/iro1::imm434 tpk1::ARG/tpk1::ARG SAT1-TAR-tetO-YAK1/SAT1-TAR-tetO-YAK1*	This study.
CaLC8412	*tetO-CYR1/CYR1*	*arg4Δ/arg4Δ his1Δ/his1Δ URA3/ura3::imm434 IRO1/iro1::imm434SAT1-TAR-tetO-CYR1/CYR1*	This study.
CaLC8414	*yak1Δ/yak1Δ*<br><br>*tetO-CYR1/CYR1*	*his1Δ/his1Δ URA3/ura3::imm434 IRO1/iro1::imm434 yak1::ARG/yak1::ARG*	This study.
CaLC8416	*efg1Δ/efg1Δ*<br><br>*tetO-YAK1/tetO-YAK1*	*arg4Δ/arg4Δ his1Δ/his1Δ URA3/ura3::imm434 IRO1/iro1::imm434 efg1::HYG/efg1::HYG SAT1-TAR-tetO-YAK1/SAT1-TAR-tetO-YAK1*	This study.
CaLC8498	*TPK2-WT tpk1Δ/tpk1Δ*<br><br>*tetO-YAK1/tetO-YAK1*	*URA3/ura3::imm434 IRO1/iro1::imm434 (tpk2::Arg)::pLC1370(TPK2WT-HIS)/(tpk2::Arg)::pLC1370(TPK2WT-HIS) tpk1::SAT1/tpk1::SAT1 HygB-TAR-tetO-YAK1/HygB-TAR-tetO-YAK1*	This study.

CaLC8499	*TPK2-AS tpk1Δ/tpk1Δ*<br><br>*tetO-YAK1/tetO-YAK1*	*URA3/ura3::imm434 IRO1/iro1::imm434 (tpk2::Arg)::pLC1370(TPK2AS-HIS)/(tpk2::Arg)::pLC1370(TPK2AS-HIS) tpk1::SAT1/tpk1::SAT1 HygB-TAR-tetO-YAK1/HygB-TAR-tetO-YAK1*	This study.
CaLC8500	*TPK2-WT tpk1Δ/tpk1Δ*<br><br>*tetO-YAK1-HF/tetO-YAK1-HF*	*URA3/ura3::imm434 IRO1/iro1::imm434 (tpk2::Arg)::pLC1370(TPK2WT-HIS)/(tpk2::Arg)::pLC1370(TPK2WT-HIS) tpk1::SAT1/tpk1::SAT1 HygB-TAR-tetO-YAK1-HF-ARG/HygB-TAR-tetO-YAK1-HF-ARG*	This study.
CaLC8502	*TPK2-AS tpk1Δ/tpk1Δ*<br><br>*tetO-YAK1-HF/tetO-YAK1-HF*	*URA3/ura3::imm434 IRO1/iro1::imm434 (tpk2::Arg)::pLC1370(TPK2AS-HIS)/(tpk2::Arg)::pLC1370(TPK2AS-HIS) tpk1::SAT1/tpk1::SAT1 HygB-TAR-tetO-YAK1-HF-ARG/HygB-TAR-tetO-YAK1-HF-ARG*	This study.
CaLC8522	*hht1Δ/hht1Δ*	*arg4Δ/arg4Δ his1Δ/his1Δ URA3/ura3::imm434 IRO1/iro1::imm434 hht1::SAT1/hht1::SAT1*	This study.
CaLC8524	*hht1Δ/hht1Δ*<br><br>*yak1Δ/yak1Δ*	*his1Δ/his1Δ URA3/ura3::imm434 IRO1/iro1::imm434 hht1::SAT1/hht1::SAT1 yak1::ARG/yak1::ARG*	This study.
CaLC8621	*FLO8/FLO8-HF-SAT1*	*arg4Δ/arg4Δ his1Δ/his1Δ URA3/ura3::imm434 IRO1/iro1::imm434 FLO8-FLO8-HF-SAT1*	This study.
CaLC8622	*FLO8/FLO8-HF-SAT1*<br><br>*yak1Δ/yak1Δ*	*his1Δ/his1Δ URA3/ura3::imm434 IRO1/iro1::imm434 FLO8-FLO8-HF-SAT1 yak1::ARG/yak1::ARG*	This study.
CaLC8675	*FLO8/FLO8-GFP-SAT1*	*arg4Δ/arg4Δ his1Δ/his1Δ URA3/ura3::imm434*	This study.

	yak1Δ/yak1Δ	*IRO1/iro1::imm434*<br>*FLO8/FLO8-GFP-SAT1*<br>*yak1::ARG/yak1::ARG*	
CaLC8702	*yak1Δ/yak1Δ* + *YAK1-HF*	*his1Δ/his1Δ URA3/ura3::imm434*<br>*IRO1/iro1::imm434*<br>*yak1::ARG/yak1::YAK1-HF-SAT1*	This study.
CaLC8704	*yak1Δ/yak1Δ* + $YAK1^{Y588F}$	*his1Δ/his1Δ URA3/ura3::imm434*<br>*IRO1/iro1::imm434*<br>*yak1::ARG/yak1::*$YAK1^{Y588F}$*-HF-SAT1*	This study.
CaLC8706	*yak1Δ/yak1Δ* + $YAK1^{S261A}$	*his1Δ/his1Δ URA3/ura3::imm434*<br>*IRO1/iro1::imm434*<br>*yak1::ARG/yak1::*$YAK1^{S261A}$*-HF-SAT1*	This study.
CaLC8753	*orf19.5253Δ/orf19.5253Δ*	*arg4Δ/arg4Δ his1Δ/his1Δ*<br>*URA3/ura3::imm434*<br>*IRO1/iro1::imm434*<br>*orf19.5253::SAT1/orf19.5253::SAT1*	This study.
CaLC8755	*orf19.5253Δ/orf19.5253Δ yak1Δ/yak1Δ*	*his1Δ/his1Δ URA3/ura3::imm434*<br>*IRO1/iro1::imm434*<br>*orf19.5253::SAT1/orf19.5253::SAT1*<br>*yak1::ARG/yak1::ARG*	This study.
CaLC8847	*yak1Δ/yak1Δ* + $YAK1^{S139A\ S212A\ S261A\ S355A\ T584A}$	*his1Δ/his1Δ URA3/ura3::imm434*<br>*IRO1/iro1::imm434*<br>*yak1::ARG/yak1::*$YAK1^{S139A\ S212A\ S261A\ S355A\ T584A}$*-HF-SAT1*	This study.
CaLC8850	*yak1Δ/yak1Δ* + tetO-$YAK1^{S139A\ S212A\ S261A\ S355A\ T584A}$	*his1Δ/his1Δ URA3/ura3::imm434*<br>*IRO1/iro1::imm434*<br>*yak1::ARG/yak1:: HygB-TAR-tetO-*$YAK1^{S139A\ S212A\ S261A\ S355A\ T584A}$*-HF-SAT1*	This study.
CaLC9128	*yak1Δ/yak1Δ* + $YAK1^{S139A}$	*his1Δ/his1Δ URA3/ura3::imm434*<br>*IRO1/iro1::imm434*<br>*yak1::ARG/yak1::*$YAK1^{S139A}$*-HF-SAT1*	This study.
CaLC9130	*yak1Δ/yak1Δ* + $YAK1^{S212A}$	*his1Δ/his1Δ URA3/ura3::imm434*<br>*IRO1/iro1::imm434*<br>*yak1::ARG/yak1::*$YAK1^{S212A}$*-HF-SAT1*	This study.
CaLC9132	*yak1Δ/yak1Δ* + $YAK1^{S139A\ S212A}$	*his1Δ/his1Δ URA3/ura3::imm434*<br>*IRO1/iro1::imm434*	This study.

		yak1::ARG/yak1::$YAK1^{S139A\ S212A}$*-HF-SAT1*	
CaLC9134	*yak1Δ/yak1Δ* + $YAK1^{S355A}$	*his1Δ/his1Δ URA3/ura3::imm434 IRO1/iro1::imm434 yak1::ARG/yak1::*$YAK1^{S355A}$*-HF-SAT1*	This study.
CaLC9136	*yak1Δ/yak1Δ* + $YAK1^{T584A}$	*his1Δ/his1Δ URA3/ura3::imm434 IRO1/iro1::imm434 yak1::ARG/yak1::*$YAK1^{T584A}$*-HF-SAT1*	This study.
CaLC9138	*yak1Δ/yak1Δ* + $YAK1^{S355A\ T584A}$	*his1Δ/his1Δ URA3/ura3::imm434 IRO1/iro1::imm434 yak1::ARG/yak1::*$YAK1^{S355A\ T584A}$*-HF-SAT1*	This study.
CaLC9144	*yak1Δ/yak1Δ* + *YAK1*	*his1Δ/his1Δ URA3/ura3::imm434 IRO1/iro1::imm434 yak1::ARG/yak1::*$YAK1^{S355A\ T584A}$*-HF-SAT1*	This study.
CaLC9146	*yak1Δ/yak1Δ* + $YAK1^{S139D\ S212D\ S261D\ S355D\ T584E}$	*his1Δ/his1Δ URA3/ura3::imm434 IRO1/iro1::imm434 yak1::ARG/yak1::*$YAK1^{S139D\ S212D\ S261D\ S355D\ T584E}$*-HF-SAT1*	This study.

Supplementary Table 2: Plasmids used in this study.

**Plasmid**	**Description**	**Source**
pLC49	p863 (for gene disruption with *FLP-NAT*)	[5]
pLC963	pV1393-1 (CaCas9/sgRNA entry expression vector, contains NatR gene, targeting *NEUT5L*)	[6]
pLC1049	CaTAR-tetOp-NAT	[7]
pLC1081	pV1093	[8]
pLC1085	pFA-6His3Flag-ARG	[9]
pLC1100	pFA-3HA-ARG	[9]
pLC1204	pFA-GFP-SAT1	This study.

pLC1210	pFA-6His3Flag-HygB	This study.
pLC1649	pMK YAK1$^{S139A\ S212A\ S261A\ S355A\ T584A}$-HF	This study.
pLC1734	pMK YAK1$^{S139D\ S212D\ S261D\ S355D\ T584E}$-HF	This study.

Supplementary Table 3: Oligonucleotides used in this study.

**Accession Number**	**Name**	**Sequence (5'-3')**
oLC274	pJK863down-F	CTGTCAAGGAGGGTATTCTGG
oLC5978	pLC963-SNR52-F	GACTGTCAAGGAGGGTATTC
oLC5979	pLC963-SNR52-N-F	CCGCAAGTGATTAGACTTAG
oLC5980	pLC963-sgRNA-R	GAATACCACTTGTTTACCGG
oLC5981	pLC963-sgRNA-N-R	GGTGGCGGCAAAACTAATTC
oLC6926	SNR52/F	AAGAAAGAAAGAAAACCAGGAGTGAA
oLC6927	sgRNA/R	ACAAATATTTAAACTCGGGACCTGG
oLC6928	SNR52/N	GCGGCCGCAAGTGATTAGACT
oLC6929	sgRNA/N	GCAGCTCAGTGATTAAGAGTAAAGATGG
oLC6942	LM21	CTAATTAACGTGTGTGTATGGATC
oLC6943	Kpp063	CACAGGATGACGCCTAAC
oLC8621	oLC8621_SNR52/R_ArgMarker	CCAAGAGCATCTCTTCTTTCCAAATTAAAAATAGTTTACGCAAGTC
oLC8622	oLC8622_sgRNA/F_ArgMarker	GAAAGAAGAGATGCTCTTGGGTTTTAGAGCTAGAAATAGCAAGTTAAA
oLC9199	oLC9199_SNR52/R_Yak1tag	CCACATTGTTATTCTTATTCCAAATTAAAAATAGTTTACGCAAGTC

oLC9200	oLC9200_SNR52/R_Yak1tag	GAATAAGAATAACAATGTGGGTTTTAGAGCTAGAAATAGCAAGTTAAA
oLC9202	oLC9202_Yak1 tag F	ACTCAATTTAAATCCATTAGAAAGATTAACACCTCAAGAAGCTTTAAAACATCCATTTATTATTGATGTTAATACCACTGATTTAGGTCGACGGATCCCC
oLC9203	oLC9203_Yak1 del/tag R	AAACAAGATTGTTACATAAACAAAATTAATTAAAAAGTATCATTAAAGAGTATAAAACTTAATACTGGTCAACCTCCCCCTCTCGATGAATTCGAGCTCG
oLC9204	oLC9204_Yak1 orf check F	CAAATTCCTGGATTCCGTAACCCTTGG
oLC9205	oLC9205_Yak1 orf check R	GCAATTCCAGCAGGACCAGAAGG
oLC9206	oLC9206_Yak1 tag check F	CAATGATTGAAAAAGAATATCATGATCGA
oLC9207	oLC9207_Yak1 tag check R	TATAAACCATATTCAGTTCTTAACAA
oLC9402	CaYAK1+611-R	TTCTTCGGTCATTACTACCC
oLC9915	CaYAK1 ApaI F -809	TTGCGGGCCCTAGAGAAAGGAAAGCAAGGG
oLC9918	CaYAK1 SacII R + 3251	TCCCCGCGGTTTAGTTGGAATTCTGGTCC
oLC10k227	YAK1 prom homology TAR F	TTAATAATATATAAGACCAACCATTGTAACCACACAAAGTATCACAGTATCACCGACAAATTTATACATAGGGCCCTTGAGATGGAGCCGTCAAATATCC
oLC10k228	YAK1 start homology tetO R	AATTGTGTCGATTAAAATTATAATTATAATTAGAACTGTTGTTATTGTTATTATATGCCATCATCCGCGGCGACTATTTATATTTGTATGTGTGTAGG
oLC10k231	YAK1 sgRNA tetO guide R	TTATGTATAAATTTGTCGGTCAAATTAAAAATAGTTTACGCAAGTC
oLC10k232	YAK1 sgRNA tetO guide F	ACCGACAAATTTATACATAAGTTTTAGAGCTAGAAATAGCAAGTTAAAA
oLC10k233	YAK1 check + 252 R	CAAGGGTTACGGAATCCAGG

oLC10k428	CaCYR1 tetO sgRNA R	CCGAAAGATAATACAGAACACAAATTAAAAATAGTTTACGCAAGTC
oLC10k429	CaCYR1 tetO sgRNA F	TGTTCTGTATTATCTTTCGGGTTTTAGAGCTAGAAATAGCAAGTTAAAA
oLC10k430	CaCYR1 tetO Repair F	TTTTCTGCATTATTGTTATTGTTTGTTGCAATTCAATTCAATTCAATTTAACCATTCATTCTCAATTTAAGGCCCTTGAGATGGAGCCGTCAAATATCC
oLC10k431	CaCYR1 tetO Repair R	GTTCCTCTAATCCAGTAGCTGAACCATCTCTAAAGTTGGCTTTAGATTTATCTCTCCTTAAAAAACTCATCATCCGCGGCGACTATTTATATTTGTATGTGTGTAGG
oLC10k432	CaCYR1 tetO WT/int test R + 82	ATTGGGTGAAAAATGAGTGG
oLC10k433	CaCYR1 tetO WT test F - 508	AGCGAATAAATAGAATCTTCGG
oLC10k562	oLC10k562_adh1 check R	GGTAGTGCTTGTTTCACAAGAAATACTATATATACTTTTATTCAATCAAGATTTTATCGATCACC
oLC10k563	CaYAK1 SDM Y588F R	GGTGATCGATAAAATCTTGATTGAATAAAAGTATATATAGTATTTCTTGTGAAACAAGCACTACC
oLC10k564	CaYAK1 SDM Piece 1 F	GGTATACTACTCAATACAATTGAAAGGGTTTCAATTATCTAGC
oLC10k565	CaYAK1 SDM Piece 2 R	CGTCCAAATGTAGATTGTAATTAGATTGATAATTATTGGG
oLC10k566	CaYAK1 SDM Fusion Outer F	GTTCAAACAAAACGCAAGAGATCACATACC
oLC10k567	CaYAK1 SDM Fusion Outer R	CTATTCCAATTCAATAGCAAGATGTAGATATTATTGAGGG
oLC10k615	Hht1 null sgRNA F	AAATCCGCCCCAGTTTCCGGGTTTTAGAGCTAGAAATAGCAAGTTAAAA
oLC10k616	Hht1 null sgRNA R	CCGGAAACTGGGGCGGATTTCAAATTAAAAATAGTTTACGCAAGTC

oLC10k617	Hht1 null SAT1 Repair F	TATAATTTAACACTACTTTCTTTCTTTCTTTCTTCTCCTTATACTTATTTAACAACACTTTTAATCAATAGGAAACAGCTATGACCATG
oLC10k618	Hht1 null SAT1 Repait R	AAACCTAAACTTAATAACATCAAACAATAATTTGTCCTAATCTATCCTATCCTATCCTATCTTATCCTGTGTAAAACGACGGCCAG
oLC10k619	Hht1 ORF check F	TCTACTGGTGGTAAAGCACC
oLC10k620	Hht1 ORF check R	GCTTCTTGTAAAGCACCAATAGC
oLC10k764	YAK1 Y588F SDM 70bp homology R	TGATAGTATTAATAAATGATTTATTGTTTTCGTCCAAATGTAGATTGTAATTAGATTGATAATTATTGGG
oLC10k765	YAK1 Y588F SDM 70bp homology F	TAAATCATTATTATTATTATTATTATTGGTATACTACTCAATACAATTGAAAGGGTTTCAATTATCTAGC
oLC10k772	YAK1 S261A SDM R	TGTTGTTGACGATAATAATTGGGTAATACCCCTACAGCTGATCTTCTATTGGCATTTACC
oLC10k773	YAK1 S261A SDM F	GGTAAATGCCAATAGAAGATCAGCTGTAGGGGTATTACCCAATTATTATCGTCAACAACA
oLC10k777	FLO8 C-term tag repair F	TTCGGGTTCAGGAGGTGCTGGCGGTACCGATGATGATAATTTCATGGGAATGAATTGGGCTGCAGATCCAATTGAAAATGGCGATGGTCGACGGATCCCC
oLC10k778	FLO8 C-term tag repair R	CAATTACTAATTTAATTGGTATTTTATTTGCTAGATCTTTACATTATCAATTTTTAAGTTGGTATGCTTGGTCATTACCAGGTCGATGAATTCGAGCTCG
oLC10k779	FLO8 C-term sgRNA F	TCAGTCAATTTATCATCTACCAAATTAAAAATAGTTTACGCAAGTC
oLC10k780	FLO8 C-term sgRNA R	GTAGATGATAAATTGACTGAGTTTTAGAGCTAGAAATAGCAAGTTAAAA
oLC10k781	FLO8 spanning check F	GAATGAATTGGGCTGCAGATCC

oLC10k782	FLO8 spanning check R	AAACTTGATTAAAAATCCTCAATCAGGTCC
oLC10k824	YAK1 sequencing Y588F F	AACTAAACAATTGTTAGATTCAATGGC
oLC10k827	YAK1 sequencing full ORF R	TGGATGTTTTAAAGCTTCTTGAGG
oLC10k828	YAK1 S261 sequencing F	CCACTCAAGAAGTTACTTTGGG
oLC10k829	YAK1 S261 sequencing R	ACAATCAGAGCTGATCTTCCC
oLC11k090	orf19.5253 sgRNA 1 R	TCAGATTCCTGGATATCGTGCAAATTAAAAATAGTTTACGCAAGTC
oLC11k091	orf19.5253 sgRNA 1 F	CACGATATCCAGGAATCTGAGTTTTAGAGCTAGAAATAGCAAGTTAAAA
oLC11k094	orf19.5253 NAT repair F	AAAAGAATACATCAAATCTTTTATACATTTGGCCCCACGTACACTGCGAACTATAGGAATCTACTAAATCGGAAACAGCTATGACCATG
oLC11k095	orf19.5253 NAT repair R	AGATTATAAATTTTCATACAGCGTATAGACGAATTGGGGAAGAAAAAAAGGGAAATTTGCTGTTTCTTGGTAAAACGACGGCCAG
oLC11k096	orf19.5253 ORF check F	CCAACCTCTACCAAAGACCG
oLC11k097	orf19.5253 ORF check R	GTTTGTTTTATGGTCGTTTGCC
oLC11k098	orf19.5253 downstream R	CGATGATCATGTGGAGAAGC
oLC11k166	Piece 1 F: 5xPKA + 5'-UTR overhang	TTAATAATATATAAGACCAACCATTGTAACCACACAAAGTATCACAGTATCACCGACAAATTTATACATAATGGCATATAATAACAATAACAACAGTTC
oLC11k167	Piece 2 R: NAT + 3-UTR overhang	TCATATTCCTATTCTTATTCCTATTCCTATTAAGAAAATTATATGATGCTATATAAACCATATTCAGTTCTATAGGAACTTCCATCAAGCTTGCC
oLC11k168	Piece 1 R: 5xPKA + NAT overhang	CTATACTGCTGTCGATTCGATACTAACGCCGCCATCCAGTGTCGACATTTTATGAT

		GGTTACTTGTCATCGTCATCTTTATAATCC
oLC11k169	Piece 2 F: NAT + 5xPKA overhang	CCATCATAAAATGTCGACACTGG
oLC11k170	Nesting primer 5xPKA F	TAAGACCAACCATTGTAACC
oLC11k171	Nesting primer NAT R	CCTATTCTTATTCCTATTCC
oLC11k320	YAK1 S355A SDM F	CCATCAACAACCTAAATATAGACGATGTGCTATAAATTCAATTCATATATCACCAGTCAATGCC
oLC11k321	YAK1 S355A SDM R	GGCATTGACTGGTGATATATGAATTGAATTTATAGCACATCGTCTATATTTAGGTTGTTGATGG
oLC11k322	YAK1 T584A SDM F	GGTAGTGCTTGTTTCACAAGAAATGCTATATATACTTATATTCAATCAAGATTTTATCGATCACC
oLC11k323	YAK1 T584A SDM R	GGTGATCGATAAAATCTTGATTGAATATAAGTATATATAGCATTTCTTGTGAAACAAGCACTACC
oLC11k324	YAK1 S355A T584A Piece 1 Fusion F	ACAATTGAAAGGGTTTCAATTATCTAGC
oLC11k325	YAK1 S355A T584A Piece 1 Fusion R	GGTGATCGATAAAATCTTGATTG
oLC11k326	YAK1 S139A SDM R	ATGTTCATAAGTTATTGGTAAATTATTTGTAAATGCTAATCGTTTATTTAGTAATGGAGG
oLC11k327	YAK1 S139A SDM F	CCATTATTACAACAACATCAACCTCCATTACTAAATAAACGATTAGCATTTACAAATAATTTACC
oLC11k328	YAK1 S212A SDM R	CCATATGGATTATAATGAGTTCCATCTACAGCAGCCGCCATTCTTCGGTCATTACTACCC
oLC11k329	YAK1 S212A SDM F	GGGTAGTAATGACCGAAGAATGGCGGCTGCTGTAGATGGAACTCATTATAATCCATATGG

oLC11k330	YAK1 S139A and S212A SDM fusion R	GGATTATAATGAGTTCCATCTACAGC

Supplementary Methods

Strain Construction

CaLC8188: C-terminally GFP tagging both *YAK1* alleles in CaLC7435 was done using a transient CRISPR method[66]. sgRNA was generated by gene specific primers oLC9199 and oLC9200 as well as universal primers oLC6926, oLC6927, oLC6928, and oLC6929 from pLC1081. The tagging cassette was amplified from pLC1204 by oLC9202 and oLC9203. Genotyping of transformants was conducted using oLC9206 and oLC9207 to verify the absence of the native *YAK1* 3'-UTR immediately downstream of the ORF.

CaLC8189: A transient CRISPR strategy was used to place both alleles of *YAK1* from CaLC2897 under the *tetO* promoter. The *NAT-tetO* repair construct was amplified from pLC1049 using primers oLC10k227 and oLC10k228. The two sgRNA components were amplified from pLC963 using primers oLC5978 and oLC10k231 as well as oLC5980 and oLC10k232. Fusion PCR was used to fuse piece 1 and piece 2 for the sgRNA using oLC5981 and oLC5979. The *NAT-tetO* repair (2 µg), sgRNA (1 µg), and Cas9 (1 µg) were transformed into CaLC6699. Integration of the *tetO* promoter was confirmed through an integration test (oLC274 and oLC10k233) as well as verification of the absence of a wild-type promoter immediately upstream of the ORF (oLC9915 and oLC9402).

CaLC8190: A transient CRISPR strategy was used to place both alleles of *YAK1* from CaLC6702 under the *tetO* promoter. The *NAT-tetO* repair construct was amplified from pLC1049 using primers oLC10k227 and oLC10k228. The two sgRNA components were amplified from pLC963

using primers oLC5978 and oLC10k231 as well as oLC5980 and oLC10k232. Fusion PCR was used to fuse piece 1 and piece 2 for the sgRNA using oLC5981 and oLC5979. The NAT-*tetO* repair (2 μg), sgRNA (1 μg), and Cas9 (1 μg) were transformed into CaLC6699. Integration of the *tetO* promoter was confirmed through an integration test (oLC274 and oLC10k233) as well as verification of the absence of a wild-type promoter immediately upstream of the ORF (oLC9915 and oLC9402).

CaLC8297: A transient CRISPR strategy was used to place both alleles of *YAK1* in CaLC6699 under the *tetO* promoter. The *NAT-tetO* repair construct was amplified from pLC1049 using primers oLC10k227 and oLC10k228. The two sgRNA components were amplified from pLC963 using primers oLC5978 and oLC10k231 as well as oLC5980 and oLC10k232. Fusion PCR was used to fuse piece 1 and piece 2 for the sgRNA using oLC5981 and oLC5979. The *NAT-tetO* repair (2 μg), sgRNA (1 μg), and Cas9 (1 μg) were transformed into CaLC6699. Integration of the *tetO* promoter was confirmed through an integration test (oLC274 and oLC10k233) as well as verification of the absence of a wild-type promoter immediately upstream of the ORF (oLC9915 and oLC9402).

CaLC8412: The *tetO* promoter was inserted upstream of the *CYR1* ORF in CaLC239 using a transient CRISPR method. sgRNA was generated by gene specific primers oLC10k428 and oLC10k429 as well as universal primers oLC5978, oLC5979, oLC5980, and oLC5981 from pLC963. The tagging cassette was amplified from pLC1049 by oLC10k430 and oLC10k431. Genotyping of transformants was conducted using oLC10k433 and oLC10k432 to verify disruption of the native *CYR1* promoter.

CaLC8414: The *tetO* promoter was inserted upstream of the *CYR1* ORF in CaLC7214 using a transient CRISPR method. sgRNA was generated by gene specific primers oLC10k428 and oLC10k429 as well as universal primers oLC5978, oLC5979, oLC5980, and oLC5981 from pLC963. The tagging cassette was amplified from pLC1049 by oLC10k430, oLC10k431. Genotyping of transformants was performed using oLC10k433 and oLC10k432 to verify the absence of the native *CYR1* promoter.

CaLC8500: *YAK1* was C-terminally His_6-$FLAG_3$-tagged in CaLC8498 using a transient CRISPR method. The two sgRNA components were amplified from pLC1081 using primers oLCoLC6926 and oLC9199 as well as oLC6927 and oLC9200. Fusion PCR was used to fuse piece 1 and piece2 for the sgRNA using oLC6928 and oLC6929. The tagging cassette was amplified from pLC1085 using oLC9202 and oLC9203. Genotyping of transformants was performed using oLC9206 and oLC9207 to verify the absence of the native *YAK1* 3'-UTR immediately downstream of the ORF.

CaLC8416: A transient CRISPR strategy was used to place both alleles of *YAK1* from CaLC8144 under the *tetO* promoter. The *NAT-tetO* repair construct was amplified from pLC1049 using primers oLC10k227 and oLC10k228. The two sgRNA components were amplified from pLC963 using primers oLC5978 and oLC10k231 as well as oLC5980 and oLC10k232. Fusion PCR was used to fuse piece 1 and piece 2 for the sgRNA using oLC5981 and oLC5979. The *NAT-tetO* repair (2 µg), sgRNA (1 µg), and Cas9 (1 µg) were transformed into CaLC6699. Integration of the *tetO* promoter was confirmed through an integration test (oLC274 and oLC10k233) as well as verifying the absence of a wild-type promoter immediately upstream of the ORF (oLC9915 and oLC9402).

CaLC8498: *YAK1* was promoter-replaced with the *tetO* promoter in CaLC6784 by a transient CRISPR method. The two sgRNA components were amplified from pLC963 using primers

oLC5978/oLC10k231 and oLC5980/oLC10k232. Fusion PCR was used to fuse piece 1 and piece 2 for the sgRNA using oLC5981 and oLC5979. The tagging cassette was amplified from pLC1031 by oLC10k558/oLC10k559. The *HYGB-tetO* repair (2 µg), sgRNA (1 µg), and Cas9 (1 µg) were transformed into CaLC6784. The oLC6393 and oLC10k233 were used to check the absence of the native YAK1 promoter.

CaLC8499: *YAK1* was promoter-replaced with the *tetO* promoter in CaLC6788 by a transient CRISPR method. The two sgRNA components were amplified from pLC963 using primers oLC5978/oLC10k231 and oLC5980/oLC10k232. Fusion PCR was used to fuse piece 1 and piece 2 for the sgRNA using oLC5981 and oLC5979. The tagging cassette was amplified from pLC1031 by oLC10k558/oLC10k559. The *HYGB-tetO* repair (2 µg), sgRNA (1 µg), and Cas9 (1 µg) were transformed into CaLC6784. oLC6393 and oLC10k233 were used to check the absence of the native YAK1 promoter.

CaLC8502: *YAK1* was C-terminally His_3-$FLAG_6$-tagged in CaLC8499 using a transient CRISPR method. The two sgRNA components were amplified from pLC1081 using primers oLCoLC6926 and oLC9199 as well as oLC6927 and oLC9200. Fusion PCR was used to fuse piece 1 and piece2 for the sgRNA using oLC6928 and oLC6929. The tagging cassette was amplified from pLC1085 by oLC9202 and oLC9203. Genotyping of transformants was performed using oLC9206 and oLC9207 were used to verify the absence of the native *YAK1* 3'-UTR immediately downstream of the ORF.

CaLC8522: A transient CRISPR strategy was used to delete both alleles of *HHT1* in CaLC239. Both sgRNA pieces were amplified from pLC963 using oLC10k615 and oLC5980 as well as oLC10k616 and oLC5978. Fusion PCR was performed to create the final sgRNA product using

oLC5979 and oLC5981. The repair cassette was amplified from pLC49 using oLC10k617 and oLC10k618. 2 µg of repair, 1 µg of sgRNA and 1 µg of Cas9 were transformed into CaLC239. oLC10k619 and oLC10k620 were used to check for the absence of a wild-type allele.

CaLC8524: A transient CRISPR strategy was used to delete both alleles of *HHT1* in CaLC7214. Both sgRNA pieces were amplified from pLC963 using oLC10k615 and oLC5980 as well as oLC10k616 and oLC5978. Fusion PCR was performed to create the final sgRNA product using oLC5979 and oLC5981. The repair cassette was amplified from pLC49 using oLC10k617 and oLC10k618. 2 µg of repair, 1 µg of sgRNA and 1 µg of Cas9 were transformed into CaLC7214. oLC10k619 and oLC10k620 were used to check for the absence of a wild-type allele.

CaLC8621: *FLO8* was C-terminally His_6-$FLAG_3$-tagged using a transient CRISPR method in CaLC239. sgRNA was generated by gene specific primers oLC10k779 and oLC10k780 as well as universal primers oLC6926, oLC6927, oLC6928, and oLC6929 from pLC1081. The repairing template was amplified from pLC1210 by oLC10k777 and oLC10k778. Transformants were genotyped using oLC10k781 and oLC10k782 to confirm an absence of the *FLO8* 3'-UTR immediately downstream of the ORF.

CaLC8622: *FLO8* was C-terminally His_6-$FLAG_3$-tagged using a transient CRISPR method in CaLC7214. sgRNA was generated by gene specific primers oLC10k779 and oLC10k780 as well as universal primers oLC6926, oLC6927, oLC6928, and oLC6929 from pLC1081. The repairing template was amplified from pLC1210 by oLC10k777 and oLC10k778. Transformants were genotyped using oLC10k781 and oLC10k782 to confirm an absence of the *FLO8* 3'-UTR immediately downstream of the ORF.

CaLC8675: *YAK1* was deleted from CaLC5654 using a transient CRISPR method. sgRNA was generated by gene specific primers oLC9199 and oLC9200 as well as universal primers oLC6926, oLC6927, oLC6928, and oLC6929 from pLC1081. The repairing template was amplified from pLC1100 by oLC9201 and 9203. Genotyping of transformants using oLC9204 and oLC9205 were used to verify *YAK1* deletion.

CaLC8702: One allele of wild-type *YAK1* was used to complement a *yak1* homozygous deletion mutant. Wild-type *YAK1* was amplified from CaLC239 using oLC10k564 and oLC10k565. An sgRNA was designed to target the ARG cassette inserted in the *YAK1* locus in the *yak1* null homozygous deletion mutant. The sgRNA was generated by gene specific primers oLC8621 and oLC8622 as well as universal primers oLC6926, oLC6927, oLC6928, and oLC6929 from pLC1081. 2 µg of repair, 1 µg of sgRNA and 1 µg of Cas9 were transformed into CaLC7214. All NATr transformants were able to grow on SC-ARG, suggesting that integration only happened to one of two yak1 deletion loci. C-terminal integration were checked by oLC6943/9918. The oLC9204/6942 amplicon were partially Sanger-sequenced by oLC10k824 and oLC10k828. Sequence covered by the two reactions matched the expected WT sequence.

CaLC8704: One allele of *YAK1* with Y588F mutated was used to complement a *yak1* homozygous deletion mutant. To create the point mutation, a fusion PCR strategy was used, with piece 1 amplified using oLC10k564 and oLC10k563 and piece 2 was amplified using oLC10k565 and oLC10k562 from CaLC239. An sgRNA was designed to target the ARG cassette inserted in the *YAK1* locus in the *yak1* null homozygous deletion mutant. The sgRNA was generated by gene specific primers oLC8621 and oLC8622 as well as universal primers oLC6926, oLC6927, oLC6928, and oLC6929 from pLC1081. 2 µg of repair, 1 µg of sgRNA and 1 µg of Cas9 were transformed into CaLC7214. All NATr transformants were able to grow on SC-ARG, suggesting

that integration only happened to one of two yak1 deletion loci. C-terminal integration were checked by oLC6943/9918. The oLC9204/6942 amplicon were partially Sanger-sequenced by oLC10k824 and oLC10k828. Sequence covered by the two reactions matched what is expected for the Y588F allele.

CaLC8706: One allele of *YAK1* with S261A mutated was used to complement a *yak1* homozygous deletion mutant. To create the point mutation, a fusion PCR strategy was used, with piece 1 amplified using oLC10k564 and oLC10k772 and piece 2 was amplified using oLC10k565 and oLC10k773 from CaLC239. An sgRNA was designed to target the ARG cassette inserted in the *YAK1* locus in the *yak1* null homozygous deletion mutant. The sgRNA was generated by gene specific primers oLC8621 and oLC8622 as well as universal primers oLC6926, oLC6927, oLC6928, and oLC6929 from pLC1081. 2 µg of repair, 1 µg of sgRNA and 1 µg of Cas9 were transformed into CaLC7214. All NATr transformants were able to grow on SC-ARG, suggesting that integration only happened to one of two yak1 deletion loci. C-terminal integration were checked by oLC6943/9918. The oLC9204/6942 amplicon were partially Sanger-sequenced by oLC10k824 and oLC10k828. Sequence covered by the two reactions matched what is expected for the S261A allele.

CaLC8753: A transient CRISPR strategy was used to delete both copies of orf19.5253 in CaLC239. Both sgRNA pieces were amplified from pLC1081 using oLC11k090/oLC6926 and oLC11k091/oLC6927. Fusion PCR was performed to create the final sgRNA product using oLC6928/oLC6929. The repair cassette was amplified from pLC49 using oLC11k094 and oLC11k095. 2 µg of repair, 1 µg of sgRNA and 1 µg of Cas9 were transformed into CaLC239. oLC11k096 and oLC11k097 were used to confirm absence of a wild-type allele and oLC11k09 and oLC274 were used to check for upstream integration of the NAT repair construct.

CaLC8755: A transient CRISPR strategy was used to delete both copies of orf19.5253 in CaLC7214. Both sgRNA pieces were amplified from pLC1081 using oLC11k090/oLC6926 and oLC11k091/oLC6927. Fusion PCR was performed to create the final sgRNA product using oLC6928/oLC6929. The repair cassette was amplified from pLC49 using oLC11k094 and oLC11k095. 2 µg of repair, 1 µg of sgRNA and 1 µg of Cas9 were transformed into CaLC239. oLC11k096 and oLC11k097 were used to confirm absence of a wild-type allele and oLC11k09 and oLC274 were used to check for upstream integration of the NAT repair construct.

CaLC8847: *YAK1* with all 5 predicted PKA phosphorylation sites phospho-ablated (S139A, S212A, S261A, S355A, T584A) was amplified from pLC1649 with primers oLC11k166 and oLC11k168. The NAT repair cassette was amplified from pLC49 with primers oLC11k169 and oLC11k167. The *YAK1* construct and NAT repair cassette then underwent fusion PCR using primers oLC11k170 and oLC11k171. The final fusion piece was then transformed into CaLC7214 using a transient CRISPR strategy with an sgRNA that targets the ARG cassette at the native *YAK1* locus in CaLC7214. oLC9206 and oLC10k764 were used to confirm downstream integration and oLC10k827 and oLC10k824 were used to check for the presence of the *YAK1* ORF. Additionally, strains were struck onto SC-ARG plates to confirm absence/presence of the ARG cassette.

CaLC8850: *YAK1* was promoter-replaced with the *tetO* promoter in CaLC8847 by a transient CRISPR method. The two sgRNA components were amplified from pLC963 using primers oLC5978/oLC10k231 and oLC5980/oLC10k232. Fusion PCR was used to fuse piece 1 and piece 2 for the sgRNA using oLC5981 and oLC5979. The tagging cassette was amplified from pLC1031 by oLC10k558/oLC10k559. The *HYGB-tetO* repair (2 µg), sgRNA (1 µg), and Cas9 (1 µg) were transformed into CaLC6784. oLC6393 and oLC10k233 were used to check the absence of the native *YAK1* promoter.

CaLC9128: One allele of *YAK1* with S139A mutated was used to complement a *yak1* homozygous deletion mutant. To create the point mutation, a fusion PCR strategy was used, with piece 1 amplified using oLC10k765 and oLC11k326 and piece 2 was amplified using oLC11k327 and oLC10k764 from CaLC239. Fusion PCR of the two pieces used primers oLC10k566 and oLC10k567. An sgRNA was designed to target the ARG cassette inserted in the *YAK1* locus in the *yak1* null homozygous deletion mutant. The sgRNA was generated by gene specific primers oLC8621 and oLC8622 as well as universal primers oLC6926, oLC6927, oLC6928, and oLC6929 from pLC1081. 2 µg of repair, 1 µg of sgRNA and 1 µg of Cas9 were transformed into CaLC7214. All NATr transformants were able to grow on SC-ARG, suggesting that integration only happened to one of two yak1 deletion loci. N-terminal integration was checked by oLC9927/10k233. The oLC10k826/6395 amplicon were partially Sanger-sequenced by oLC9156, oLC10k824, oLC10k828, oLC10k829, and oLC11k324. Sequence covered by the sequencing reactions matched what is expected for the S139A allele.

CaLC9130: One allele of *YAK1* with S212A mutated was used to complement a *yak1* homozygous deletion mutant. To create the point mutation, a fusion PCR strategy was used, with piece 1 amplified using oLC10k765 and oLC11k328 and piece 2 was amplified using oLC11k329 and oLC10k764 from CaLC239. Fusion PCR of the two pieces used primers oLC10k566 and oLC10k567. An sgRNA was designed to target the ARG cassette inserted in the *YAK1* locus in the *yak1* null homozygous deletion mutant. The sgRNA was generated by gene specific primers oLC8621 and oLC8622 as well as universal primers oLC6926, oLC6927, oLC6928, and oLC6929 from pLC1081. 2 µg of repair, 1 µg of sgRNA and 1 µg of Cas9 were transformed into CaLC7214. All NATr transformants were able to grow on SC-ARG, suggesting that integration only happened to one of two yak1 deletion loci. N-terminal integration was checked by oLC9927/10k233. The

oLC10k826/6395 amplicon were partially Sanger-sequenced by oLC9156, oLC10k824, oLC10k828, oLC10k829, and oLC11k324. Sequence covered by the sequencing reactions matched what is expected for the S212A allele.

CaLC9132: One allele of *YAK1* with S139A and S212A mutated was used to complement a *yak1* homozygous deletion mutant. To create the point mutations, a three-part fusion PCR strategy was used, with piece 1 amplified using oLC10k765 and oLC11k326, piece 2 was amplified using oLC11k327 and oLC11k328, and piece 3 was amplified using oLC11k329 and oLC10k764 from CaLC239. Fusion PCR for piece 1 and piece 2 used primers oLC10k566 and oLC11k328 and this piece was fused with piece 3 using oLC10k566 and oLC10k567. An sgRNA was designed to target the ARG cassette inserted in the *YAK1* locus in the *yak1* null homozygous deletion mutant. The sgRNA was generated by gene specific primers oLC8621 and oLC8622 as well as universal primers oLC6926, oLC6927, oLC6928, and oLC6929 from pLC1081. 2 µg of repair, 1 µg of sgRNA and 1 µg of Cas9 were transformed into CaLC7214. All NATr transformants were able to grow on SC-ARG, suggesting that integration only happened to one of two yak1 deletion loci. N-terminal integration was checked by oLC9927/10k233. The oLC10k826/6395 amplicon were partially Sanger-sequenced by oLC9156, oLC10k824, oLC10k828, oLC10k829, and oLC11k324. Sequence covered by the sequencing reactions matched what is expected for the S139A S212A allele.

CaLC9134: One allele of *YAK1* with S355A mutated was used to complement a *yak1* homozygous deletion mutant. To create the point mutation, a fusion PCR strategy was used, with piece 1 amplified using oLC10k765 and oLC11k321 and piece 2 was amplified using oLC11k320 and oLC10k764 from CaLC239. Fusion PCR of the two pieces used primers oLC10k566 and oLC10k567. An sgRNA was designed to target the ARG cassette inserted in the *YAK1* locus in the

yak1 null homozygous deletion mutant. The sgRNA was generated by gene specific primers oLC8621 and oLC8622 as well as universal primers oLC6926, oLC6927, oLC6928, and oLC6929 from pLC1081. 2 µg of repair, 1 µg of sgRNA and 1 µg of Cas9 were transformed into CaLC7214. All NATr transformants were able to grow on SC-ARG, suggesting that integration only happened to one of two yak1 deletion loci. N-terminal integration was checked by oLC9927/10k233. The oLC10k826/6395 amplicon were partially Sanger-sequenced by oLC9156, oLC10k824, oLC10k828, oLC10k829, and oLC11k324. Sequence covered by the sequencing reactions matched what is expected for the S355A allele.

CaLC9136: One allele of *YAK1* with T584A mutated was used to complement a *yak1* homozygous deletion mutant. To create the point mutation, a fusion PCR strategy was used, with piece 1 amplified using oLC10k765 and oLC11k321 and piece 2 was amplified using oLC11k322 and oLC10k764 from CaLC239. Fusion PCR of the two pieces used primers oLC10k566 and oLC10k567. An sgRNA was designed to target the ARG cassette inserted in the *YAK1* locus in the *yak1* null homozygous deletion mutant. The sgRNA was generated by gene specific primers oLC8621 and oLC8622 as well as universal primers oLC6926, oLC6927, oLC6928, and oLC6929 from pLC1081. 2 µg of repair, 1 µg of sgRNA and 1 µg of Cas9 were transformed into CaLC7214. All NATr transformants were able to grow on SC-ARG, suggesting that integration only happened to one of two yak1 deletion loci. N-terminal integration was checked by oLC9927/10k233. The oLC10k826/6395 amplicon were partially Sanger-sequenced by oLC9156, oLC10k824, oLC10k828, oLC10k829, and oLC11k324. Sequence covered by the sequencing reactions matched what is expected for the S212A allele.

CaLC9138: One allele of *YAK1* with S355A and T584A mutated was used to complement a *yak1* homozygous deletion mutant. To create the point mutations, a three-part fusion PCR strategy was

used, with piece 1 amplified using oLC10k765 and oLC11k321, piece 2 was amplified using oLC11k320 and oLC11k323, and piece 3 was amplified using oLC11k322 and oLC10k764 from CaLC239. Fusion PCR for piece 1 and piece 2 used primers oLC10k566 and oLC11k323 and this piece was fused with piece 3 using oLC10k566 and oLC10k567. An sgRNA was designed to target the ARG cassette inserted in the *YAK1* locus in the *yak1* null homozygous deletion mutant. The sgRNA was generated by gene specific primers oLC8621 and oLC8622 as well as universal primers oLC6926, oLC6927, oLC6928, and oLC6929 from pLC1081. 2 µg of repair, 1 µg of sgRNA and 1 µg of Cas9 were transformed into CaLC7214. All NATr transformants were able to grow on SC-ARG, suggesting that integration only happened to one of two yak1 deletion loci. N-terminal integration was checked by oLC9927/10k233. The oLC10k826/6395 amplicon were partially Sanger-sequenced by oLC9156, oLC10k824, oLC10k828, oLC10k829, and oLC11k324. Sequence covered by the sequencing reactions matched what is expected for the S355A T584A allele.

CaLC9144: One allele of wild-type *YAK1* was used to complement a *yak1* homozygous deletion mutant. Wild-type *YAK1* was amplified from CaLC155 using oLC10k564 and oLC10k565. An sgRNA was designed to target the ARG cassette inserted in the *YAK1* locus in the *yak1* null homozygous deletion mutant. The sgRNA was generated by gene specific primers oLC8621 and oLC8622 as well as universal primers oLC6926, oLC6927, oLC6928, and oLC6929 from pLC1081. 2 µg of repair, 1 µg of sgRNA and 1 µg of Cas9 were transformed into CaLC7214. All NATr transformants were able to grow on SC-ARG, suggesting that integration only happened to one of two yak1 deletion loci. C-terminal integration were checked by oLC6943/9918. The oLC9204/6942 amplicon were partially Sanger-sequenced by oLC10k824 and oLC10k828. Sequence covered by the two reactions matched the expected WT sequence.

CaLC9146: SC5314 phospho-mimetic *YAK1* with all 5 predicted PKA phosphorylation sites mutated to phospho-mimetic residues (S139D, S212D, S261D, S355D, T584E) was amplified from pLC1734 with primers oLC11k166 and oLC11k168. The NAT repair cassette was amplified from pLC49 with primers oLC11k169 and oLC11k167. The *YAK1* construct and NAT repair cassette then underwent fusion PCR using primers oLC11k170 and oLC11k171. The final fusion piece was then transformed into CaLC7214 using a transient CRISPR strategy with an sgRNA that targets the ARG cassette at the native *YAK1* locus in CaLC7214. oLC9206 and oLC10k764 were used to confirm downstream integration and oLC10k827 and oLC10k824 were used to check for the presence of the *YAK1* ORF. Additionally, strains were struck onto SC-ARG plates to confirm absence/presence of the ARG cassette.

References

1. Gillum, A. M., Tsay, E. Y., & Kirsch, D. R. Isolation of the *Candida albicans* gene for orotidine-5'-phosphate decarboxylase by complementation of *S. cerevisiae* ura3 and *E. coli* pyrF mutations. *Mol. Gen. Genet.* **198**, 179-182 (1984).
2. Noble, S. M., & Johnson, A. D. Strains and strategies for large-scale gene deletion studies of the diploid human fungal pathogen *Candida albicans*. *Eukaryot. Cell* **4**, 298-309 (2005).
3. Xie, J. L., O'Meara, T. R., Polvi, E. J., Robbins, N., & Cowen, L. E. Staurosporine induces filamentation in the human fungal pathogen *Candida albicans* via signaling through Cyr1 and Protein Kinase A. *mSphere* **2**, e00056-17 (2017).
4. Polvi, E. J., et al. Functional divergence of a global regulatory complex governing fungal filamentation. *PLoS Genet.* **15**, e1007901 (2019).
5. Shen, J., Guo, W., & Kohler, J. R. CaNAT1, a heterologous dominant selectable marker for transformation of *Candida albicans* and other pathogenic *Candida* species. *Infect. Immun.* **73**, 1239-1242 (2005).

6. Veri, A. O., et al. Tuning Hsf1 levels drives distinct fungal morphogenetic programs with depletion impairing Hsp90 function and overexpression expanding the target space. *PLoS Genet.* **14**, e1007270 (2018).

7. Kim, S. H. et al., Genetic analysis of *Candida auris* implicates Hsp90 in morphogenesis and azole tolerance and Cdr1 in azole resistance. *mBio* **10**, e00346-19 (2019).

8. Min, K., Ichikawa, Y., Woolford, C. A., & Mitchell, A. P. *Candida albicans* gene deletion with a transient CIRSPR-Cas9 system. *mSphere* **1**, e00130-16 (2016).

9. Zhang, A., Petrov, K. O., Hyun, E. R., Liu, Z., Gerber, S. A., and Myers, L. C. The Tlo proteins are stoichiometric components of *Candida albicans* mediator anchored via the Med3 subunit. *Eukaryot. Cell* **11**, 874-884 (2012).
